# Load carriage physiology in normoxia and hypoxia

**DOI:** 10.1007/s00421-023-05320-2

**Published:** 2023-09-23

**Authors:** Daniel A. Baur, Katherine G. Baur, Beverley K. Buchanan, Miles J. Ortiz, Abaigeal G. Doody

**Affiliations:** Department of Human Performance and Wellness, Virginia Military Institute, 208 Cormack Hall, Lexington, VA 24450 USA

**Keywords:** Near-infrared spectroscopy, Hemodynamics, Altitude, Ventilation, Carbohydrate oxidation

## Abstract

**Purpose:**

To determine the effects of load carriage in normoxia and normobaric hypoxia on ventilatory responses, hemodynamics, tissue oxygenation, and metabolism.

**Methods:**

Healthy males (n = 12) completed 3 randomly ordered baseline graded exercise tests in the following conditions: (1) unloaded normoxic (U: F_I_O_2_ = 20.93%), (2) loaded (~ 30 kg) normoxic (LN), and (3) loaded hypoxic simulating ~ 3650 m (LH: F_I_O_2_ =  ~ 13%). Thereafter, experimental exercise trials were completed in quasi-randomized order (i.e., U completed first) consisting of 3 × 10 min of walking (separated by 5 min seated rest) with stages matched with the U condition (in ascending order) for relative intensity, absolute oxygen consumption ([VO_2_]; 1.7 L min^−1^), and walking speed (1.45 ± 0.15 m s^−1^).

**Results:**

Load carriage increased perceived exertion and reduced VO_2max_ (LN: − 7%; LH: − 32%; *p* < 0.05). At matched VO_2_, stroke volume and tidal volume were reduced and maintained with LN and LH vs. U, respectively (*p* < 0.05). Increases in cardiac output and minute ventilation at matched VO_2_ (with LH) and speed (with LN and LH), were primarily accomplished via increases in heart rate and breathing frequency (*p* < 0.05). Cerebral oxygenated hemoglobin (O_2_HHb) was increased at all intensities with LN, but deoxygenated hemoglobin and total hemoglobin were increased with LH (*p* < 0.05). Muscle oxygen kinetics and substrate utilization were similar between LN and U, but LH increased CHO dependence and reduced muscle O_2_HHb at matched speed (*p* < 0.05).

**Conclusion:**

Load carriage reduces cardiorespiratory efficiency and increases physiological strain, particularly in hypoxic environments. Potential load carriage-induced alterations in cerebral blood flow may increase the risk for altitude illnesses and requires further study.

## Introduction

Thoracic load carriage is an occupational requirement for many populations including search and rescue, military personnel, and wildland firefighters (Knapik et al. 2004; Sol et al. 2018; Faghy et al. 2022). While necessary to fulfill operational duties, load carriage impairs exercise capacity and task performance (Knapik et al. 1991; Holewun and Lotens 2007; Drain et al. 2016). Thus, research investigating the physiological impact of thoracic load carriage is warranted to develop countermeasures, improve technology, and ensure health and safety.

Decades of research have improved our understanding of load carriage physiology. The effects of load carriage on energy expenditure are well-documented, and numerous modeling equations have been developed to aid in operational planning (Pandolf et al. 1977; Santee et al. 2001; Looney et al. 2018). In addition to energy cost, research has established that load carriage increases physiological strain, as recently reviewed (Faghy et al. 2022). For example, load carriage has been reported to induce a shallower breathing pattern (i.e., reduced tidal volume [V_T_] due to a reduced end inspiratory lung volume and increased breathing frequency [f_B_]) leading to increased dead space ventilation (Phillips et al. 2016b). Moreover, prolonged load carriage has also been reported to cause respiratory muscle fatigue (Faghy and Brown 2014; Armstrong et al. 2019). Nevertheless, few studies have investigated physiological responses to load carriage when matched for oxygen demand with the unloaded condition (Phillips et al. 2016b, c; Shei et al. 2018). As such, it is challenging to determine the degree to which effects reported in prior studies stem from the load or the difference in exercise intensity resulting from load carriage matched for walking speed with the unloaded condition. In addition, it is yet to be determined whether the increased respiratory muscle work attendant to load carriage, which can modulate the respiratory muscle pump mechanism and cause a metaboreflex (Miller et al. 2005; Dempsey et al. 2006), redirects blood flow or alters oxygenation of the brain or locomotor muscles. If so, this has potentially negative implications for performance and cognitive function.

There is also limited research on the effects of load carriage in high altitudes. This is surprising given the well-described health risks and performance decrements that occur (Young and Reeves 2002), and the fact that load carriage in hypoxic environments is commonplace due to the impracticality or impossibility of mechanized resupply in mountainous terrain (Knapik et al. 2004). A study by Hinde et al. (2018) investigated the effects of load (18.2 kg), hypoxia (~ 11.8% fraction of inspired oxygen [F_I_O_2_]), and cold (− 10 °C) on ventilatory responses and respiratory muscle fatigue. Similar to the findings of others in normoxic conditions (Phillips et al. 2016b), load carriage in hypoxia resulted in increased minute ventilation (V_E_) and f_B_ as well as reduced V_T_. This increase in ventilatory work likely contributed to the respiratory muscle fatigue observed following exercise. In a follow-up study (Hinde et al. 2020), respiratory muscle fatigue was similarly observed following a 6-km loaded walk in hypoxia (18.2 kg; 50% VO_2max_ in hypoxia [~ 11.8% F_I_O_2_]) regardless of inspiratory muscle training. Importantly, cerebral and muscle tissue oxygenation were not assessed in either study preventing evaluation of potential alterations in blood flow, which may be more likely in hypoxia owing to the increased respiratory muscle load, and more impactful due to the heightened competition for blood/oxygen in this environment. Of interest, Rosales et al. (2022) recently reported reduced brain, but maintained muscle, oxygenation with unloaded stepping exercise in normobaric or hypobaric hypoxia; however, it is unknown whether these responses are influenced by load carriage.

Similarly, questions remain regarding the hemodynamic and metabolic effects of thoracic load carriage in normoxia or hypoxia. Theoretically, the mechanical compression of the chest and shoulders induced by load carriage may impair cardiac preload, stroke volume (SV), and cardiac output (Q) in ways similar to chest wall restriction with inelastic straps (Miller et al. 2002). However, research evaluating responses to actual load carriage is equivocal and complicated by methodological inconsistency between studies (Sagiv et al. 2006; Nelson et al. 2009). Interpretation of metabolic data is challenging for similar reasons. A number of recent studies, including one that employed load carriage as the exercise mode (Griffiths et al. 2019a), have established that carbohydrate (CHO) oxidation in hypoxia is mediated by intensity with similar responses to normoxia when matched for relative intensity and increases when matched for absolute oxygen consumption (VO_2_) (Young et al. 2018; Griffiths et al. 2019b). However, it remains to be determined whether load carriage itself influences metabolism independent of environmental context. This is due to the fact that most load carriage studies employ designs that prevent detection of load carriage-specific effects, which may be expected given the above-described physiological strain imposed (Faghy et al. 2022). Specifically, most prior load carriage studies that reported metabolic data either did not include an unloaded comparison condition (Griffiths et al. 2019a) or made comparisons to unloaded exercise based on matched walking speeds (Blacker et al. 2009; Faghy et al. 2016), which results in unequal VO_2_ between conditions. Few studies measured metabolic responses based upon matched oxygen demand (Phillips et al. 2016b, c), and no studies accounted for the reduction in VO_2max_ induced by load carriage (Phillips et al. 2016c) or hypoxia (Ferretti et al. 1997) and matched for relative intensity (%VO_2max_). Thus, more research is warranted to determine load carriage-specific hemodynamic and metabolic effects and whether effects are mediated by hypoxia across the most physiologically (i.e., %VO_2max_ and absolute VO_2_) and practically (i.e., walking speed) important intensities.

Therefore, the purpose of this study was to determine the physiological responses to load carriage in normoxia and hypoxia. Specifically, the study assessed changes in ventilatory mechanics, hemodynamics, tissue oxygenation, and metabolism.

## Materials and methods

### Study design and ethical approval

This study employed a quasi-randomized and single-blinded design to assess the physiological impact of load carriage in normoxia and hypoxia. For each subject, the study encompassed ~ 2 weeks, included 1 visit to the Post infirmary (described below), and 6 total laboratory visits consisting of: (1–3) baseline testing and (4–6) experimental trials. Baseline testing and experimental trials were conducted in the following conditions: (1) unloaded normoxic (U; 20.93% F_I_O_2_), (2) loaded normoxic (LN; 20.93% F_I_O_2_), and (3) loaded hypoxic (LH; ~ 13.0% F_I_O_2_). For the loaded conditions, subjects wore a Modular Lightweight Load-Carrying Equipment (MOLLE) pack loaded to 29.5 kg with cotton packing material, ~ 20 kg sandbag, and weight plates. The load was packed to be positioned high in the pack and close to the body. The mass was chosen because it corresponds with the typical fighting load carried by combat soldiers (Dean 2004). In the LH condition, normobaric hypoxic generators (Everest II, Hypoxico, Gardiner, NY, USA) were utilized to reduce F_I_O_2_ to ~ 13.0%, which is equivalent to ~ 3650 m altitude when accounting for laboratory elevation (~ 325 m), barometric pressure (~ 738 mmHg), and 47 mmHg water vapor pressure (Conkin 2011). The baseline testing conditions were randomized. For the experimental trials, all subjects completed the U condition first, which permitted close matching of subsequent conditions for the prescribed intensities. The final 2 experimental trials were randomized. All visits were separated by ≥ 48 h.

For the Post infirmary visit, blood was drawn from an antecubital vein and tested for the sickle cell trait, which can increase the risk of complications in hypoxic environments and was therefore exclusionary (Goodman et al. 2014). During this initial visit, informed consent was obtained from subjects following a full description of study requirements. All study protocols were approved by the Virginia Military Institute Institutional Review Board.

### Subjects

Healthy males (n = 12; age = 21 ± 1 years; height = 179.5 ± 6.7 cm; mass = 81.7 ± 11.2 kg; body fat (%) = 13.7 ± 4.8%) were recruited from Virginia Military Institute and the surrounding community to participate in this study. Due to technical difficulties with measurements for 2 subjects, cerebral oxygenation and hemodynamics are presented as n = 10. Subjects were recreationally active (i.e., participating in aerobic or resistance training ≥ 3 times per week) and had a range of load carriage experience. Exclusion criteria included: smoking, presence of heart disease, BMI > 30, orthopedic issues that would have prevented them from completing the activities required for the study, sickle cell trait, and recent altitude exposure (i.e., had traveled to ≥ 1500 m within the last 3 months).

### Baseline testing

For the initial visit, subjects reported to the laboratory in athletic clothing (t-shirt and shorts) and running shoes. Height, weight, and body composition were assessed via stadiometer, electronic scale, and air displacement plethysmography (BodPod, Cosmed, Inc., Rome, Italy), respectively. For subsequent baseline testing visits, all procedures were identical with the exception of body composition analysis (which was not repeated).

Following initial measurements, subjects completed an incremental exercise test consisting of 4–5 × 3-min stages at 8% gradient designed to establish VO_2_/speed relationships for each subject. During testing, VO_2_ was measured constantly via a breath-by-breath metabolic analysis system calibrated according to manufacturer’s instructions (Metalyzer 3B, Cortex, Leipzig, Germany). This testing was completed with or without a pack depending on condition and included a range of walking speeds to encompass all potential speeds that elicited VO_2_ values needed to match intensities between conditions (described below). Based on data from these tests, individualized walking speeds were prescribed for each subject for use in experimental trials. If necessary, these speeds were later adjusted based on real-time VO_2_ responses within the first 4 min of experimental trial testing.

Following this test, subjects rested for ~ 5 min prior to commencing an incremental exercise test to volitional exhaustion for determination of maximal oxygen consumption (VO_2max_). The protocol for this test differed based on the condition (i.e., U, LN, or LH). Distinct protocols were developed in response to pilot testing, which revealed that a standardized protocol resulted in localized muscle fatigue/early exercise cessation in certain conditions due to large discrepancies in test duration. Thus, in order to reduce duration variability and increase the likelihood of VO_2max_ attainment, subjects completed the following incremental protocols based on condition: (U) exercise commenced at 9.7 km h^−1^ and 1% gradient; each min, the speed was increased by 1.6 km h^−1^ until 12.9 km h^−1^ was reached; thereafter, speed was held constant and the gradient was increased 1% each min until volitional exhaustion; (LN) exercise commenced at 6.4 km h^−1^ and 0% gradient for 1 min; thereafter, the gradient was increased to 4% for 1 min and increased a further 1% each min until volitional exhaustion; (LH) exercise commenced at 6.4 km h^−1^ and 0% gradient; thereafter, the gradient was increased 1% until volitional exhaustion. Gas exchange was assessed continuously during testing. Ratings of perceived exertion (RPE) were measured in the final 20 s of each stage, and blood lactate (Lactate Plus, Nova Biomedical, Waltham, MA, USA) was measured 3 min following test cessation. Attainment of a “true” VO_2max_ (i.e., highest 30-s average) was based on fulfillment of ≥ 2 of the following criteria: plateau in VO_2_ in the final exercise stage, respiratory exchange ratio (RER) ≥ 1.15, heart rate (HR) within 10 b min^−1^ of age predicted maximum (220-age), RPE ≥ 19, and/or blood lactate ≥ 8 mmol L^−1^ (Howley et al. 1995). Out of 36 completed tests, 34 were deemed a “true” VO_2max_. Given the preponderance of subjects meeting the criteria for all tests, the authors felt justified in using this data for calculation of relative intensity for experimental trials (described below) and employing the term “VO_2max_” (i.e., rather than VO_2peak_) hereafter in describing study results.

### Experimental trials

**Fig. 1:**
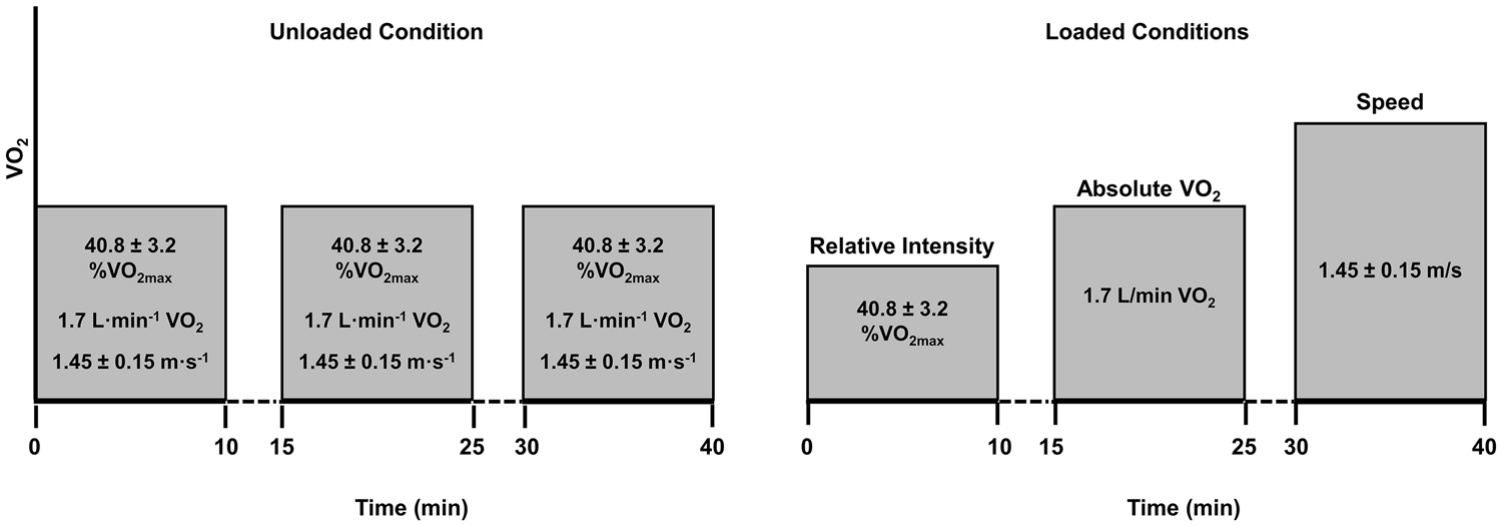
Overview of experimental exercise protocols

The experimental exercise protocol is presented in Fig. 1. Subjects arrived at the laboratory in the morning (0700–1000) following an overnight fast (8–10 h) and were weighed. Following measurement preparation procedures (e.g., placement of electrodes), subjects entered an enclosed air-tight chamber and rested quietly for 10 min. Thereafter, resting measurements were collected for 5 min. Subjects then commenced the exercise protocol. The protocol was designed to allow for the matching of physiologically and practically important intensities with the U condition. In the U condition, subjects completed 3 × 10 min stages while walking at 8% gradient and a speed that elicited a VO_2_ of 1.7 L min^−1^. This VO_2_ was targeted as it elicited a walking pace consistent with prior research and military doctrine (Pihlainen et al. 2014; Young et al. 2018; TRADOC 2022). Additionally, it was sufficiently low-intensity to permit completion of 10-min stages in the hypoxic condition given the expected large decreases in VO_2max_ in this condition (and consequent increases in relative intensity; Ferretti et al. 1997). Each stage was separated by 5 min of seated rest. In the loaded conditions (i.e., LN and LH), subjects similarly completed 3 × 10 min stages at 8% gradient. However, the speeds employed in each stage were customized for each subject in order to match the relative intensity (%VO_2max_; stage 1), absolute VO_2_ (1.7 L min^−1^; stage 2), and walking speed (stage 3) of the U condition.

### Cardiovascular responses

Hemoglobin saturation (SpO_2_) was measured continuously via pulse oximeter (Wrist Ox_2_ Model 3150, Nonin, Plymouth, MN, USA). Hemodynamic variables (i.e., Q, HR, SV, end diastolic volume [EDV], end systolic volume [ESV], ejection fraction [EF], and systemic vascular resistance [SVR]), were measured via impedance cardiagraphy (Physioflow Enduro, Manatec Biomedical, Poissy, France). These measurements required the placement of six electrodes on the abdomen and neck based on manufacturer’s instructions. Electrodes were affixed following site prep with alcohol swabs and abrasive gel (Nuprep, Weaver and Company, Aurora, CO, USA) to remove dead skin. Following the first experimental trial, electrode sites were marked with a permanent marker to ensure consistent placement for remaining trials. Measurements were taken continuously, and the final 3 min of each exercise stage were averaged for analysis.

### Oxygen kinetics

Near-infrared spectroscopy (NIRS) was employed to assess skeletal muscle (Portamon, Artinis Medical Systems, Elst, The Netherlands) and cerebral (Octamon, Artinis Medical Systems, Elst, The Netherlands) oxygenated (O_2_HHb) and deoxygenated hemoglobin (HHb). Total hemoglobin (tHHb), which is an index for regional blood flow (Van Beekvelt et al. 2001), represented the sum of O_2_HHb and HHb. For muscle oxygenation, a small area on the right lateral gastrocnemius was shaved prior to securing the device via black-colored elastic wrap, which prevented entry of extraneous light. Following the first experimental trial, the device site was marked with a permanent marker to ensure consistent placement between trials. Data was recorded at 10 Hz and with a differential pathlength factor of 4.94 in accordance with others (Duncan et al. 1995; Shannon et al. 2017). For cerebral oxygenation, the device containing 8 light emitters and 2 light detectors was worn by the subject over their forehead (1 cm above the eyebrows) and provided measures for the pre-frontal cortex. For this device, the differential pathlength factor was adjusted based on the subject’s age (Duncan et al. 1995). Both devices employ dual wavelengths (760 and 850 nm) to measure oxygen kinetics as previously described based on hemoglobin’s absorbance and scattering of near infrared light at these wavelengths (Boushel and Piantadosi 2000; Neary 2004). Tissue oxygenation was measured continuously and averaged over the final 3 min of each stage. Data were analyzed as the change in O_2_HHb, HHb, and tHHb relative to baseline values obtained following 5 min seated rest in normoxia prior to entering the enclosed chamber.

### Gas exchange and blood metabolites

Expired gases and ventilatory responses were assessed via a breath-by-breath gas analysis system as described above. Absolute CHO and fat oxidation were calculated via application of stoichiochemical equations to gas exchange results on the assumption that protein oxidation is negligible during exercise (Jeukendrup and Wallis 2005). Relative CHO and fat oxidation were derived from RER measurements as follows:

$$\% {\text{CHO}}\, = \,\left( {\left( {RER{-}0.7} \right)/0.3} \right) \, \times 100$$

$$\% {\text{Fat}}\, = \,100 - \% CHO.$$

This equation applies to the metabolic respiratory quotient and yields the relative energetic contribution of CHO and fat oxidation, expressed in energy equivalents. Blood glucose (Cardiochek Plus, PTS Diagnostics, Whitestown, IN, USA) and lactate were measured following fingerstick blood draws by portable devices calibrated based on manufacturer’s instructions. Gas exchange was assessed continuously, and data were averaged over the final 3 min of each stage. Blood metabolites were measured at the end of each stage.

### Perceptual responses

RPE was assessed in the last 30 s of each stage via Borg scale (6–20) (Borg 1982).

### Control procedures

Baseline testing was completed following ≥ 2 h fast and 24 h with no exercise. Experimental trials were completed in the morning (0700–1000) following an overnight fast (8–10 h) and after abstaining from exercise for 24 h. In addition, subjects were asked to maintain consistent dietary and exercise habits throughout the study, which were confirmed by review of completed 24-h diet logs and 72-h exercise logs. To control for pre-experimental trial nutrition, subjects were advised to consume a meal consisting of pasta and tomato sauce the evening prior.

### Statistical analysis

Means and standard deviations (SD) are presented for all dependent measures. Responses for hemodynamics, ventilatory variables, metabolism, and oxygen kinetics were analyzed via two-way repeated measures analysis of variance (ANOVA) to identify main effects and interactions. Residuals produced by the ANOVA were assessed for approximate normality via Shapiro–Wilk test and visual inspection. Effect sizes for ANOVAs were calculated as partial eta squared (${\eta }_{p}^{2})$ (Cohen 1973). Post hoc simple contrasts were employed in the case of a significant interaction to identify differences between conditions at the various time points. Maximal exercise data were analyzed via one-way ANOVA with post hoc Tukey tests to identify significant differences. All post hoc testing was conducted with Bonferroni adjustment for multiple comparisons. For post hoc testing results, effect sizes (Cohen’s *d*) were calculated by standardizing mean differences to the pooled SD of the three conditions as follows: ≤ 0.2, trivial, ≥ 0.2 small, > 0.6 moderate, and > 1.2 large (Cohen 2013). The sample size of 12 subjects was selected because it aligned with prior studies of load carriage (Phillips et al. 2016b; Hinde et al. 2018). Additionally, prior studies employing similar designs or equipment (Phillips et al. 2016b; Rosales et al. 2022) observed effect sizes for V_T_ and SV of *d* ≥ 0.9, which would be detected with sufficient statistical power (1 − β = 0.8) given the sample used in the present study (n = 12). All analyses were performed using IBM SPSS Statistics (Version 29), and the α level for statistical significance was set at 0.05.

## Results

### Maximal exercise

Maximal exercise responses are presented in Table 1. Compared to U, VO_2max_ (L min^−1^) was reduced with LN (*d* = 0.98; *p* < 0.001) and LH (*d* = 4.31; *p* < 0.001); LH was also lower than LN (*d* = 3.4; *p* < 0.001). Similarly, maximal heart rate (HR_max_) was reduced in the loaded conditions vs. U (*d* = 0.74–1.4; *p* < 0.01), and there was a trend for a difference between LH and LN (*d* = 0.65; *p* = 0.052). There were also small (*d* = 0.53; *p* < 0.001) and medium (*d* = 0.80; *p* < 0.001) reductions in V_T_ at VO_2max_ with LN and LH vs. U, respectively, but no differences existed between loaded conditions. f_B_ at VO_2max_ was increased with LH vs. U (*d* = 0.51; *p* = 0.021), but there were no differences between loaded conditions. Finally, blood lactate was reduced with LH vs. U at VO_2max_ (*d* = 0.71; *p* = 0.047).

**Table 1:** Maximal exercise responses

	U	LN	LH
VO_2max_ (L min^−1^)	4.3 (0.4)^a^	3.9 (0.4)^b^	2.9 (0.2)
VO_2max_ (mL kg^−1^ min^−1^)	52.5 (4.9)^a^	48.8 (5.6)^b^	35.3 (3.5)
HR_max_ (b min^−1^)	195.2 (6.6)^a^	189.3 (7.9)	184.2 (9.1)
V_E_ (L min^−1^)	159.4 (8.6)	152.6 (15.6)	152.5 (14.1)
V_T_ (L)	2.9 (0.4)^a^	2.7 (0.3)	2.6 (0.4)
f_B_ (b min^−1^)	56.1 (7.0)^b^	57.3 (5.4)	60.0 (9.5)
Lactate (mmol L^−1^)	12.3 (3.2)^c^	10.3 (2.2)	11.4 (2.8)
SpO_2_ (%)	89.2 (2.7)^b^	91.1 (2.0)^d^	70.2 (2.7)

Data is presented as mean (SD). *VO*_*2max*_ maximal oxygen consumption, *HR* heart rate, *V*_*E*_ minute ventilation, *V*_*T*_ tidal volume, *f*_*B*_ breathing frequency, *SpO*_*2*_ oxygen saturation, *U* unloaded normoxic, *LN* loaded normoxic, *LH* loaded hypoxic. ^a^Denotes different vs. LN and LH (*p* < 0.05); ^b^denotes different vs. LH (*p* < 0.05); ^c^denotes different vs. LN (*p* < 0.05); ^d^denotes different vs. U and LH (*p* < 0.05)

### Oxygen consumption, walking speed, environmental conditions, and perceived exertion

These data are presented in Table 2. Generally, results for VO_2_, relative intensity, walking speed, and F_I_O_2_ confirmed the study design. Temperature (21.4 ± 1.4 °C; *p* = 0.12) and humidity (51.2 ± 9.9%; *p* = 0.67) were also confirmed to be consistent across conditions. Perceived exertion was increased by load carriage across all exercise stages (*d* = 0.9–2.5; *p* < 0.05). Moreover, perceived exertion was increased with LH vs. LN during exercise matched for absolute VO_2_ (*d* = 1.3; *p* = 0.002) and speed (*d* = 1.0; *p* < 0.001).

**Table 2:** Selected data representing exercise intensities, environmental conditions, and perceptual responses during exercise at intensities matched to the unloaded condition for relative VO_2max_, absolute VO_2_, and walking speed

	U	LN	LH
VO_2_ (L min^−1^)			
Relative	1.69 (0.05)	1.56 (0.12)^a^	1.15 (0.08)^b^
Absolute	1.73 (0.08)	1.73 (0.08)*	1.74 (0.06)*
Speed	1.73 (0.08)	2.45 (0.27)*	2.30 (0.17)*^a^
Relative intensity (%VO_2max_)			
Relative	40.0 (3.6)	39.7 (3.4)	40.2 (3.1)
Absolute	40.8 (3.6)	44.1 (4.2)*^a^	60.9 (4.6)*^b^
Speed	40.9 (3.3)	62.6 (10.4)*^a^	80.9 (9.7)*^b^
Walking speed (m s^−1^)			
Relative	1.4 (0.2)	0.9 (0.2)^a^	0.6 (0.1)^b^
Absolute	1.4 (0.2)	1.0 (0.2)*^a^	1.0 (0.1)*^a^
Speed	1.4 (0.2)	1.4 (0.2)*	1.4 (0.2)*
Perceived exertion (6–20)			
Relative	7.7 (0.9)	8.8 (1.2)^a^	9.3 (1.7)^a^
Absolute	8.2 (0.8)	9.6 (1.5)*^a^	11.3 (1.7)*^b^
Speed	8.1 (0.9)	11.5 (2.4)*^a^	13.9 (3.1)*^b^
F_i_O_2_ (%; exercise mean)	20.93 (0.004)	20.93 (0.003)	12.95 (0.048)^b^

Progressive exercise stages were designed to match conditions at the following intensities: *relative* VO_2_ (%VO_2max_; stage 1), *absolute* VO_2_ (L min^−1^; stage 2), and walking *speed* (m s^−1^; stage 3). Data is presented as mean (SD). *VO*_*2*_ oxygen consumption, *VO*_*2max*_ maximal oxygen consumption, *F*_*i*_*O*_*2*_ fraction of inspired oxygen, *U* unloaded, *LN* loaded normoxic, *LH* loaded hypoxic. *Denotes different from the lower intensity (*p* < 0.05); ^a^denotes different vs. U (*p* < 0.05); ^b^denotes different vs. U and LN (*p* < 0.05)

### Ventilatory responses

**Fig. 2:**
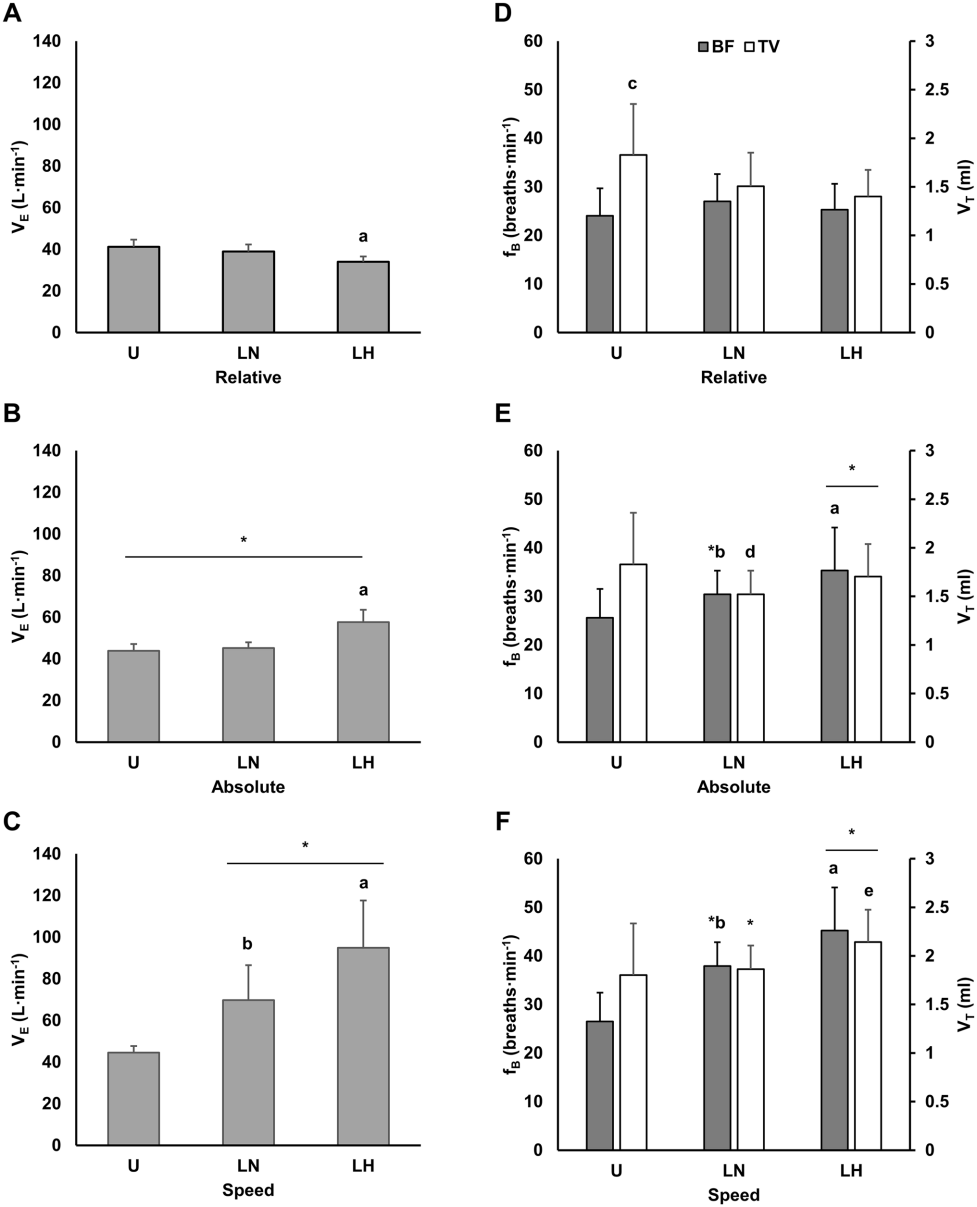
**A**–**C**, ventilation (V_E_) and **D**–**F**, breathing frequency (f_B_) and tidal volume (V_T_) responses at intensities matched to the unloaded condition for relative VO_2max_ (40%VO_2max_), absolute VO_2_ (1.7 L min^−1^), and walking speed (1.4 m s^−1^). respectively. All data is presented as mean ± SD. *U* unloaded, *LN* loaded normoxic, *LH* loaded hypoxic. *Denotes different from the lower intensity (*p* < 0.05); **a** denotes different vs. U and LN (*p* < 0.05); **b** denotes different vs. U (*p* < 0.05); **c** denotes different vs. LN and LH (*p* < 0.01); **d** denotes different vs. U and LH (*p* < 0.05); **e** denotes different vs. LN (*p* < 0.001)

Ventilation data are presented in Fig. 2. For V_E_ and f_B_, there were condition (${\eta }_{p}^{2}$ = 0.73–0.86; *p* < 0.001), time (${\eta }_{p}^{2}$ = 0.90–0.94; *p* < 0.001), and interaction effects (${\eta }_{p}^{2}$ = 0.73–0.83; *p* < 0.001). For V_T_, there were time (${\eta }_{p}^{2}$ = 0.86; *p* < 0.001) and interaction effects (${\eta }_{p}^{2}$ = 0.61; *p* < 0.001).

At rest, V_E_ was increased with LH (12.0 ± 2.5 L min^−1^) vs. U (10.1 ± 1.6 L min^−1^; *d* = 0.96; *p* = 0.003) and LN (10.4 ± 1.5 L min^−1^; *d* = 0.79; *p* = 0.004); however, there were no differences in V_T_ (U: 0.8 ± 0.3 L; LN: 0.9 ± 0.3 L; LH: 1.0 ± 0.4 L) or f_B_ (U: 13.9 ± 3.7 b min^−1^; LN: 13.5 ± 3.7 b min^−1^; LH: 13.9 ± 4.1 b min^−1^) across conditions.

At matched relative intensity, there were large reductions in V_E_ with LH compared to U (*d* = 2.30; *p* < 0.001) and LN (*d* = 1.56; *p* < 0.001). Additionally, V_T_ was higher with U vs. both loaded conditions (LH: *d* = 0.82–1.08; *p* < 0.01).

At the same absolute VO_2_, there were large increases V_E_ with LH compared to U (*d* = 3.27; *p* < 0.001) and LN (*d* = 2.93; *p* < 0.001). Moreover, there were medium (*d* = 0.79) and small (*d* = 0.47) reductions in V_T_ in the LN condition compared to U (*p* = 0.027) and LH (*p* < 0.001), respectively. This coincided with a medium (*d* = 0.71) increase in f_B_ with LN vs. U (*p* = 0.003). Finally, f_B_ was increased with LH compared to the other conditions (*d* = 0.73–1.44; *p* < 0.05).

When matched for walking speed, there were stepwise increases in V_E_ and f_B_ across conditions. Specifically, there were large and medium increases in V_E_ (*d* = 1.54; *p* < 0.001) and f_B_ (*d* = 1.18; *p* < 0.001), respectively, with LN vs. U. Additionally, V_E_ and f_B_ were elevated with LH vs. U (*d* = 1.94–3.10; *p* < 0.001) and LN (*d* = 0.77–1.54; *p* < 0.01). V_T_ increased in the LH condition relative to LN (*d* = 0.70; *p* < 0.001), and there was a tendency for a medium (*d* = 0.86) increase vs. U (*p* = 0.063). However, there were no differences in V_T_ between the normoxic conditions.

### Hemodynamics

**Fig. 3:**
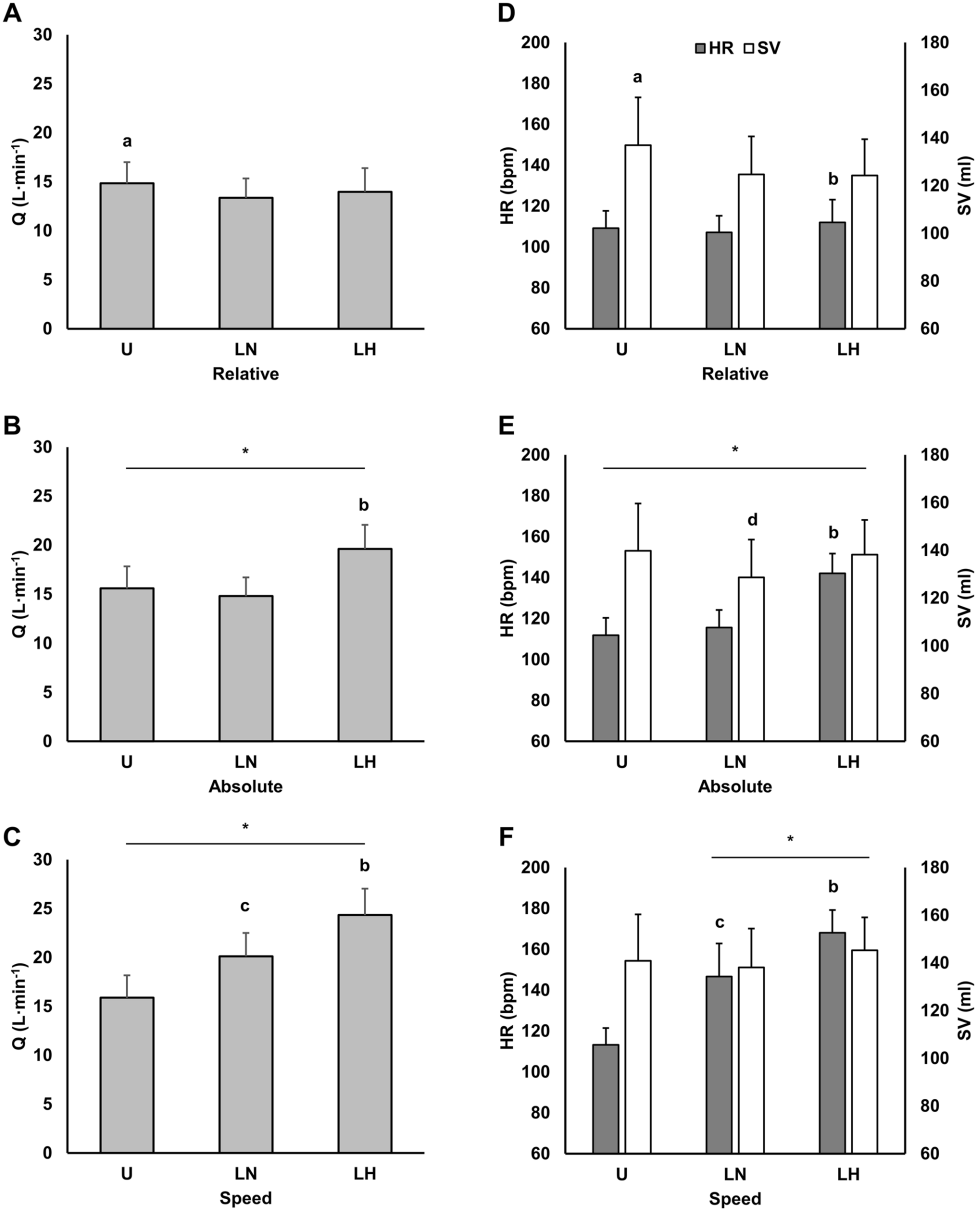
**A**–**C** Cardiac output (Q) and **D**–**F** heart rate (HR) and stroke volume (SV) responses at intensities matched to the unloaded condition for relative VO_2max_ (40%VO_2max_), absolute VO_2_ (1.7 L min^−1^), and walking speed (1.4 m s^−1^), respectively. All data is presented as mean ± SD. *U* unloaded, *LN* loaded normoxic, *LH* loaded hypoxic. *Denotes different from the lower intensity (*p* < 0.05); **a** denotes different vs. LN and LH (*p* < 0.05); **b** denotes different vs. U and LN (*p* < 0.01); **c** denotes different vs. U (*p* < 0.001); **d** denotes different vs. U and LH (*p* < 0.05)

Cardiovascular responses are presented in Fig. 3 and Table 3. For Q, HR, SVR, and SpO_2_ there were condition (${\eta }_{p}^{2}$ = 0.73–0.98; *p* < 0.001), time (${\eta }_{p}^{2}$ = 0.91–0.98; *p* < 0.001), and interaction effects (${\eta }_{p}^{2}$ = 0.60–0.91; *p* < 0.001). Additionally, there were time (${\eta }_{p}^{2}$ = 0.93–0.98) and interactions effects (${\eta }_{p}^{2}$ = 0.32–0.68) for SV (*p* < 0.001), EDV (*p* < 0.001), and EF (*p* < 0.01). ESV changed over time (${\eta }_{p}^{2}$ = 0.34; *p* = 0.026), but there were no condition or interaction effects. For SpO_2_, there were large reductions with LH versus the normoxic conditions (*d* = 5.85–11.50; *p* < 0.001) at rest and for all exercise stages. Additionally, there was a small reduction in SpO_2_ with LN vs. U when matched for walking speed (*d* = 0.46; *p* < 0.001).

**Table 3:** Selected hemodynamic variables during exercise at intensities matched to the unloaded condition for relative VO_2max_ (40%VO_2max_), absolute VO_2_ (1.7 L min^−1^), and walking speed (1.4 m s^−1^)

	U	LN	LH
EDV (mL)			
Relative	196.2 (36.4)^a^	179.4 (24.3)	176.4 (26.2)
Absolute	194.3 (29.4)	182.2 (25.1)^b^	188.8 (27.1)*
Speed	195.0 (29.8)	189.2 (24.9)*	196.6 (24.8)*
ESV (mL)			
Relative	59.2 (25.2)	54.7 (19.8)	52.2 (17.2)
Absolute	54.5 (20.3)	53.6 (20.6)	50.6 (19.0)*
Speed	54.2 (20.3)	51.2 (19.6)*	51.3 (17.3)*
EF (%)			
Relative	70.7 (7.6)	70.3 (8.2)	70.8 (5.9)
Absolute	72.3 (7.4)	70.9 (8.3)	73.9 (6.7)*
Speed	72.9 (7.4)	73.6 (7.6)*	74.8 (6.2)*
SVR (dyn s^−1^ cm^−5^)			
Relative	470.7 (77.1)	513.6 (91.3)	504.6 (100.1)
Absolute	448.5 (72.1)*	460.0 (74.7)*	353.5 (51.8)*^c^
Speed	441.6 (72.7)	338.5 (51.8)*^b^	283.1 (36.8)*^c^
SpO_2_ (%)			
Relative	94.9 (2.7)	95.2 (1.1)	78.0 (3.9)^c^
Absolute	95.2 (1.1)	94.5 (2.0)	74.0 (3.5)*^c^
Speed	94.8 (0.8)	93.9 (0.8)^b^	72.6 (3.1)^c^

Data is presented as mean (SD). *EDV* end diastolic volume, *EF* ejection fraction, *ESV* end systolic volume, *SVR* systemic vascular resistance, *SpO*_*2*_ hemoglobin saturation, *U* unloaded, *LN* loaded normoxic, *LH* loaded hypoxic. *Denotes different from the lower intensity (*p* < 0.05); ^a^denotes different vs. LN and LH (*p* < 0.05); ^b^denotes different vs. U (*p* < 0.01); ^c^denotes different vs. U and LN (*p* < 0.01)

At rest, Q was increased with LH (7.2 ± 1.0 L min^−1^) compared to U (6.1 ± 1.1 L min^−1^; *d* = 1.11; *p* < 0.008) and LN (6.1 ± 0.8 L min^−1^; *d* = 1.10; *p* = 0.008). Similarly, HR was increased with LH (74.4 ± 7.1) versus the other conditions (U: 65.3 ± 7.4, *d* = 1.35; *p* < 0.001; LN: 64.7 ± 5.7, *d* = 1.44; *p* = 0.006). Finally, there were large (*d* = 1.34; *p* = 0.004) and medium (*d* = 0.90; *p* = 0.008) reductions in SVR with LH (942.7 ± 149.6 dyn s^−1^ cm^−5^) versus LN (1091.3 ± 151.7 dyn s^−1^ cm^−5^) and U (1162.6 ± 189.0 dyn s^−1^ cm^−5^), respectively.

At matched relative intensity, Q and SV were increased with U vs. LN (*d* = 0.68–0.71; *p* < 0.01) and LH (*d* = 0.40–0.74; *p* < 0.05), but there were no differences between loaded conditions. Similarly, EDV was increased with U vs. the loaded conditions (*d* = 0.57–0.67; *p* < 0.05).

At matched absolute VO_2_, Q and HR were elevated with LH compared to U (*d* = 1.82–3.41; *p* < 0.001) and LN (*d* = 2.17–2.99; *p* < 0.001). These differences coincided with large (*d* = 1.42–1.59) reductions in SVR with LH relative to the other conditions (*p* < 0.001). In addition, there were medium (*d* = 0.66) and small (*d* = 0.56) reductions in SV with LN vs. U (*p* = 0.011) and LH (*p* = 0.014), respectively. This coincided with a small reduction in EDV with LN vs. U (*d* = 0.44; *p* < 0.001). There were no differences in SV or EDV between U and LH.

When matched for walking speed, there were stepwise increases in Q and HR across conditions, with LN higher than U (*d* = 1.72–2.71; *p* < 0.001) and LH higher than both U (*d* = 2.72–3.43; *p* < 0.001) and LN (*d* = 1.71–1.74; *p* < 0.01). These differences were mirrored by stepwise reductions in SVR across conditions. Specifically, there were large reductions with LN (*d* = 1.85; *p* = 0.002) and LH (*d* = 2.84; *p* < 0.001) vs. U and a medium decrease with LH vs. LN (*d* = 0.99; *p* = 0.002).

### Muscle oxygen kinetics

**Fig. 4:**
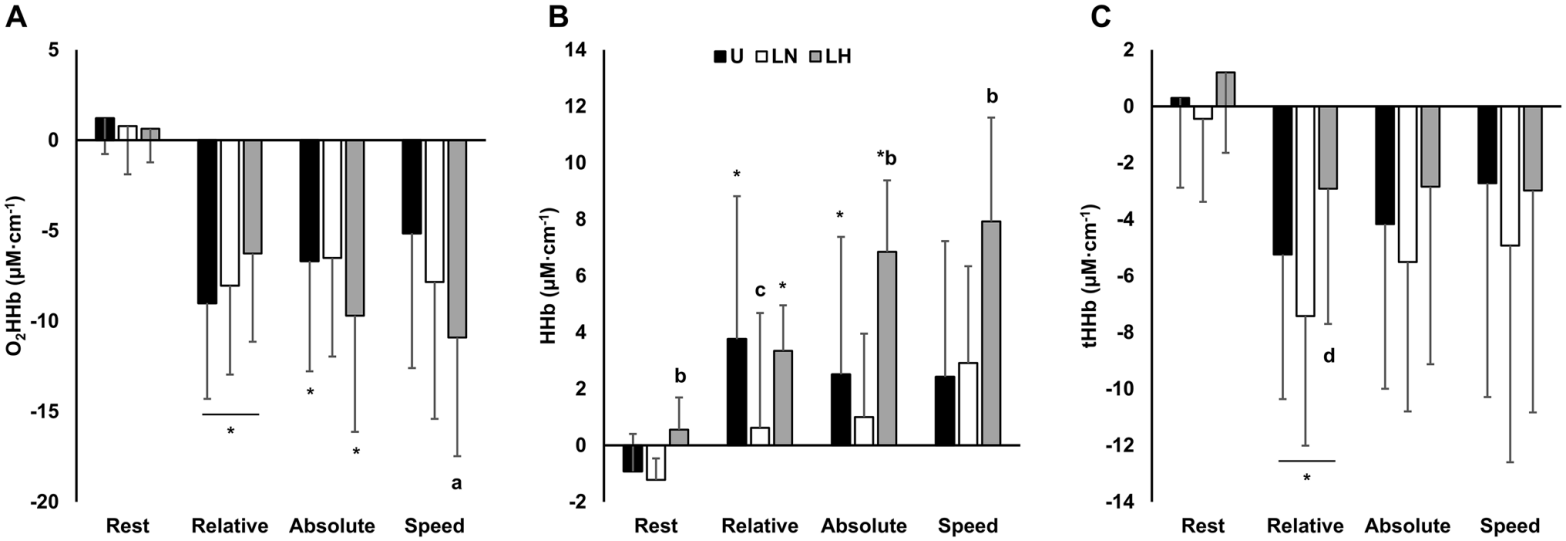
The change in muscle oxyhemoglobin (O_2_HHb) (**A**), deoxyhemoglobin (HHb) (**B**), and total hemoglobin (tHHb) concentrations (**C**) at intensities matched to the unloaded condition for relative VO_2max_ (40%VO_2max_), absolute VO_2_ (1.7 L min^−1^), and walking speed (1.4 m·s^−1^), respectively. All data is presented as mean ± SD. U, unloaded; LN, loaded normoxic; LH, loaded hypoxic. *Denotes different from the lower intensity (*p* < 0.05); **a** denotes different vs. U (*p* < 0.05); **b** denotes different vs. U and LN (*p* < 0.05); **c** denotes different vs. U and LH (*p* < 0.05); **d** denotes different vs. LN (*p* = 0.032)

Muscle O_2_HHb, HHb, and tHHb are presented in Fig. 4. There was a main effect of time (${\eta }_{p}^{2}$ = 0.68; *p* < 0.001) and an interaction effect (${\eta }_{p}^{2}$ = 0.43; *p* < 0.001) for muscle O_2_HHb. For muscle HHb, there were condition (η^2^ = 0.54; *p* < 0.001), time (${\eta }_{p}^{2}$ = 0.61; *p* < 0.001), and interaction effects (${\eta }_{p}^{2}$ = 0.49; *p* < 0.001). For tHHb, there was a main effect of time (${\eta }_{p}^{2}$= 0.465; *p* = 0.006), but no condition or interaction effects.

At rest, there were no differences in muscle O_2_HHb or tHHb. However, there were large increases in muscle HHb with LH relative to U (*d* = 1.34; *p* = 0.032) and LN (*d* = 1.62; *p* < 0.001).

When matched for relative intensity, muscle O_2_HHb was similar between conditions. However, there were medium reductions in muscle HHb with LN vs. U (*d* = 0.81; *p* = 0.024) and LH (*d* = 0.71; *p* = 0.049). Muscle HHb was similar between U and LH. Additionally, tHHb was increased with LH vs. LN (*d* = 0.93*; p* = 0.032) but was similar to U.

At the same absolute VO_2_, there were no differences in muscle O_2_HHb across conditions, but there were large increases in muscle HHb with LH relative to U (*d* = 1.21; *p* = 0.002) and LN (*d* = 1.63; *p* < 0.001). Muscle HHb was similar between normoxic conditions. There were also no differences between conditions for tHHb.

At the same walking speed, muscle O_2_HHb and HHb were similar between normoxic conditions. However, muscle O_2_HHb was reduced with LH vs. U (*d* = 0.80; *p* = 0.015). Moreover, there were large increases in muscle HHb with LH compared to U (*d* = 1.37; *p* = 0.01) and LN (*d* = 1.25; *p* = 0.003). tHHb was similar between conditions when matched for speed.

### Cerebral oxygen kinetics

**Fig. 5:**
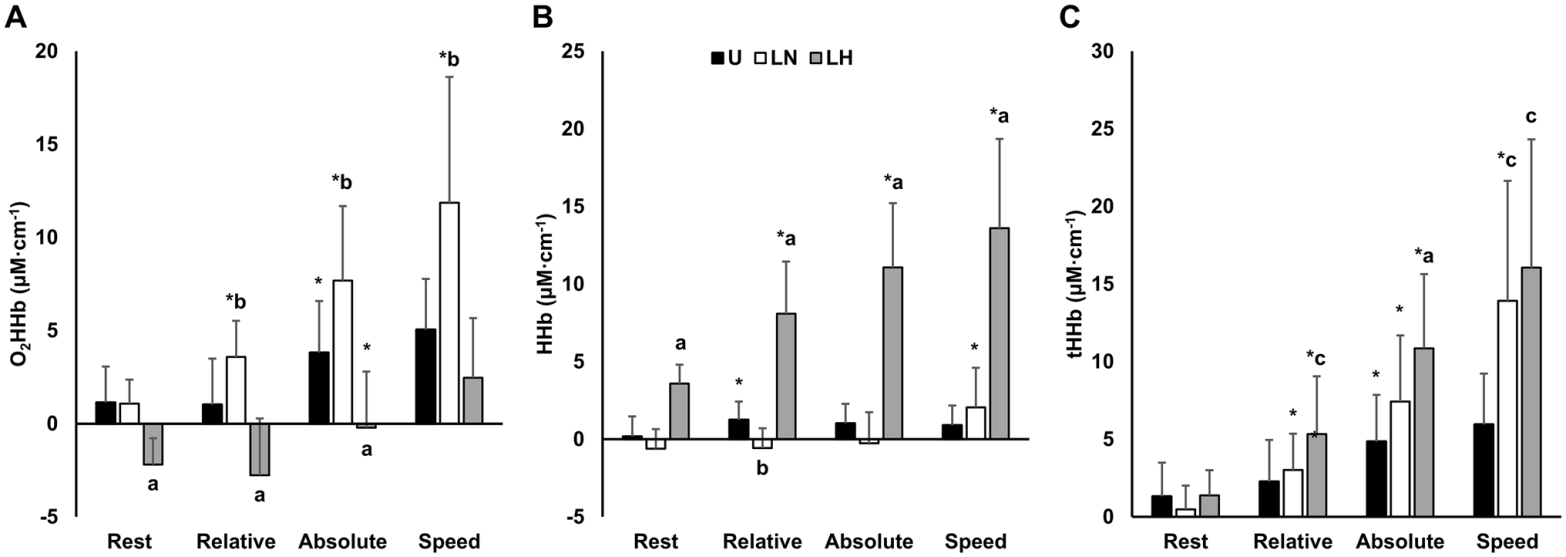
The change in cerebral oxyhemoglobin (O_2_HHb) (**A**), deoxyhemoglobin (HHb) (**B**), and total hemoglobin (tHHb) concentrations (**C**) at intensities matched to the unloaded condition for relative VO_2max_ (40%VO_2max_), absolute VO_2_ (1.7 L min^−1^), and walking speed (1.4 m s^−1^); respectively. All data is presented as mean ± SD. *U* unloaded, *LN* loaded normoxic, *LH* loaded hypoxic. *Denotes different from the lower intensity (*p* < 0.05); **a** denotes different vs. U and LN (*p* < 0.05); **b** denotes different vs. U and LH (*p* < 0.05); **c** denotes different vs. U (*p* < 0.05)

Cerebral O_2_HHb, HHb, and tHHb are presented in Fig. 5. Condition (${\eta }_{p}^{2}$ = 0.76; *p* < 0.001), time (${\eta }_{p}^{2}$ = 0.76; *p* < 0.001), and interaction effects (${\eta }_{p}^{2}$ = 0.42; *p* < 0.001) were observed for cerebral O_2_HHb. Similarly, there were condition (${\eta }_{p}^{2}$ = 0.77; *p* < 0.001), time (${\eta }_{p}^{2}$ = 0.91; *p* < 0.001), and interaction effects (${\eta }_{p}^{2}$ = 0.75; *p* < 0.001) present for cerebral HHb. Finally, there were condition (${\eta }_{p}^{2}$ = 0.51; *p* = 0.002), time (${\eta }_{p}^{2}$ = 0.84; *p* < 0.001), and interaction effects (${\eta }_{p}^{2}$ = 0.51; *p* = 0.001) for tHHb.

At rest in the LH condition, cerebral O_2_HHb and HHb were decreased and increased, respectively, when compared to U (*d* = 2.1–2.7; *p* < 0.01) and LN (*d* = 2.1–3.3; *p* < 0.001). There were no differences in tHHb between conditions at rest.

When matched for relative intensity, cerebral O_2_HHb was reduced with LH relative to U (*d* = 1.50; *p* = 0.008) and LN (*d* = 2.51; *p* < 0.001), and there were corresponding large increases in cerebral HHb with LH relative to U (*d* = 3.13; *p* < 0.001) and LN (*d* = 3.97; *p* < 0.001). Conversely, there was a medium increase and decrease in O_2_HHb (*d* = 1.01; *p* = 0.046) and HHb (*d* = 0.84; *p* = 0.006), respectively, with LN vs. U. Additionally, there was a medium increase in tHHb with LH relative to U (*d* = 1.0; *p* = 0.023) but no difference vs. LN.

At the same absolute VO_2_, there were large reductions in cerebral O_2_HHb with LH versus the normoxic conditions (*d* = 1.22–2.39; *p* < 0.01), which were mirrored by large increases in HHb (*d* = 3.64–4.10; *p* < 0.001). Conversely, there was a medium (*d* = 1.17) increase in cerebral O_2_HHb with LN vs. U (*p* = 0.026), but there was no difference in HHb. For tHHb, there were increases with LH compared to U (*d* = 1.5; *p* = 0.006) and LN (*d* = 0.84; *p* = 0.038), and there was a strong trend for an increase with LN vs. U (*d* = 0.63; *p* = 0.051).

Finally, cerebral O_2_HHb was similar between LH and U at the same walking speed. However, there were large increases in HHb with LH compared to the normoxic conditions (*d* = 3.11–3.42; *p* < 0.001). Additionally, there were large increases in O_2_HHb with LN vs. U (*d* = 1.48; *p* = 0.025) and LH (*d* = 2.04; *p* = 0.003). However, HHb was similar between LN and U (*p* = 0.29). tHHb was increased in both loaded conditions relative to U (*d* = 1.17–1.49; *p* < 0.05) but was similar between LN and LH.

### Metabolism

**Fig. 6:**
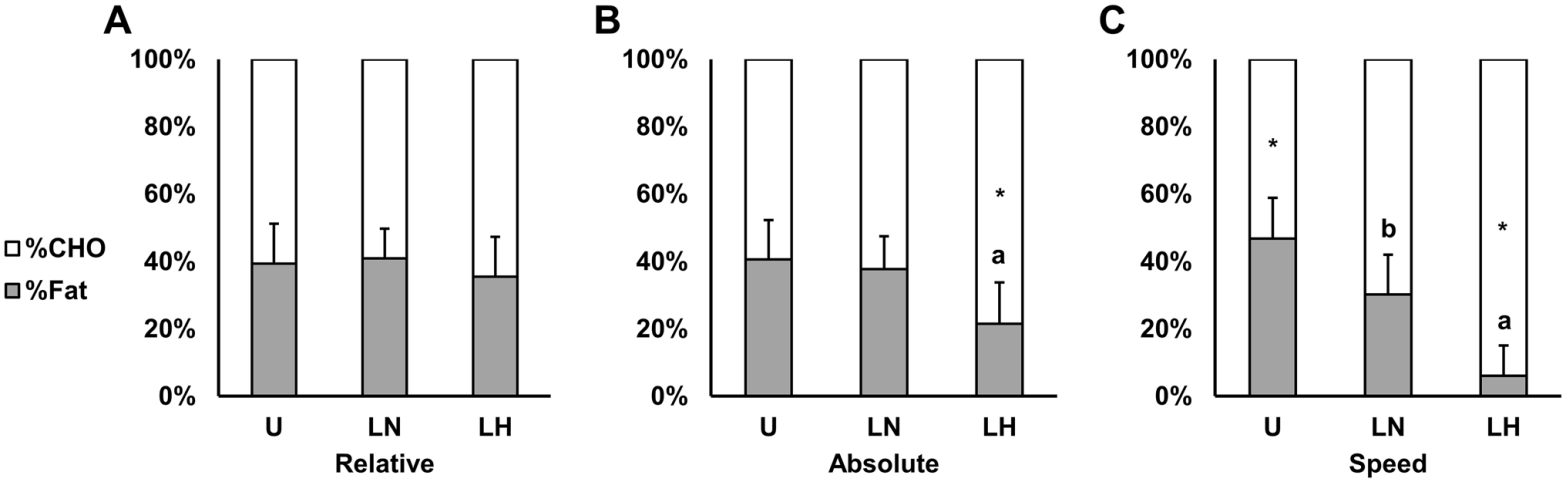
Relative (%) contribution of carbohydrate and fat oxidation at intensities matched to the unloaded condition for relative VO_2max_ (**A**; 40.0%VO_2max_), absolute VO_2_ (**B**; 1.7 L min^−1^), and walking speed (**C**; 1.4 m s^−1^), respectively. All data is presented as mean ± SD. *CHO* carbohydrate, *U* unloaded, *LN* loaded normoxic, *LH* loaded hypoxic. *Denotes different from the lower intensity (*p* < 0.05); **a** denotes different CHO and fat oxidation vs. U and LN (*p* < 0.001); **b** denotes different CHO and fat oxidation vs. U (*p* < 0.001)

Metabolic responses are presented in Fig. 6 and Table 4. There were condition, time, and interaction effects observed for absolute CHO and fat oxidation (${\eta }_{p}^{2}$ = 0.71–0.88; *p* < 0.001), relative CHO and fat oxidation (${\eta }_{p}^{2}$ = 0.45–0.73; *p* < 0.01), and blood lactate (${\eta }_{p}^{2}$ = 0.61–0.87; *p* ≤ 0.001). Additionally, there were condition (${\eta }_{p}^{2}$ = 0.64; *p* < 0.001) and interaction effects (${\eta }_{p}^{2}$ = 0.29; *p* < 0.001) present for blood glucose, but no main effect of time.

**Table 4:** Substrate oxidation, blood glucose, and lactate responses to exercise at intensities matched to the unloaded condition for relative VO_2max_ (40%VO_2max_), absolute VO_2_ (1.7 L min^−1^), and walking speed (1.4 m s^−1^)

	U	LN	LH
CHO oxidation (g min^−1^)			
Relative	1.3 (0.3)	1.1 (0.2)	0.9 (0.2)^a^
Absolute	1.3 (0.3)	1.3 (0.2)*	1.7 (0.3)*^a^
Speed	1.1 (0.3)*	2.1 (0.6)*^b^	3.1 (0.8)*^a^
Fat oxidation (g min^−1^)			
Relative	0.3 (0.1)	0.3 (0.1)	0.2 (0.1)^a^
Absolute	0.4 (0.1)	0.3 (0.1)	0.2 (0.1)^a^
Speed	0.4 (0.1)*	0.4 (0.1)	0.1 (0.1)*^a^
Blood glucose (mg dL^−1^)			
Relative	101.8 (7.1)	105.2 (7.1)	115.3 (9.6)^a^
Absolute	103.2 (8.4)	105.1 (8.2)	113.4 (8.5)^a^
Speed	103.1 (3.5)	105.4 (7.9)	124.1 (16.8)^a^
Blood lactate (mmol L^−1^)			
Relative	1.1 (0.4)	1.0 (0.2)	1.5 (0.2)^a^
Absolute	0.9 (0.4)	1.2 (0.4)	2.5 (0.6)*^a^
Speed	0.9 (0.5)	2.2 (1.5)^b^	6.0 (2.6)*^a^

Data is presented as mean (SD). *CHO* carbohydrate, *U* unloaded, *LN* loaded normoxic, *LH* loaded hypoxic. *Denotes different from the lower intensity (*p* < 0.05); ^a^denotes different vs. U and LN (*p* < 0.05); ^b^denotes different vs. U (*p* < 0.01)

At rest, there were increases and decreases in absolute CHO oxidation and fat oxidation, respectively, with LH (CHO: 0.43 ± 0.16; fat: 0.03 ± 0.03) vs. U (CHO: 0.25 ± 0.09; fat: 0.08 ± 0.02; *d* = 1.4–2.0; *p* < 0.001) and LN (CHO: 0.28 ± 0.12; fat: 0.08 ± 0.02; *d* = 1.0–1.9; *p* < 0.01). This corresponded with increases and decreases (*d* = 1.8–2.2; *p* < 0.01) in relative CHO (U: 51.8 ± 13.6%; LN: 56.4 ± 14.8%; 83.7 ± 15.2%) and fat oxidation (U: 48.2 ± 13.6%; LN: 43.6 ± 14.8%; LH: 16.3 ± 15.2%), respectively. There were no differences between U and LN for fuel selection at rest. There were additionally no differences in blood glucose or lactate between conditions at rest.

At the same relative intensity, absolute CHO and fat oxidation were reduced with LH relative to U (*d* = 1.54–1.66; *p* < 0.01) and LN (*d* = 1.04–1.38; *p* < 0.05). However, there were no differences between any condition for relative CHO or fat oxidation. There were also large increases in blood glucose and lactate with LH compared to U (*d* = 1.36–1.69; *p* < 0.01) and LN (*d* = 1.26–1.62; *p* < 0.05).

Matched for absolute VO_2_, there were large (*d* = 1.34–1.74) increases and decreases in CHO and fat oxidation (i.e., relative and absolute), respectively, between LH and the normoxic conditions (*p* < 0.01). Similarly, blood glucose (*d* = 0.99–1.22) and lactate (*d* = 2.82–3.39) were both increased with LH vs. the other conditions (*p* < 0.05). There were no differences between any of these variables with LN compared to U.

At the same walking speed, absolute and relative CHO oxidation were increased with LN vs. U (*d* = 1.48–1.72; *p* < 0.01), and with LH vs. U (*d* = 3.32–3.66; *p* < 0.001) and LN (*d* = 1.61–2.18; *p* < 0.001). Absolute and relative fat oxidation were correspondingly reduced with LH vs. U (*d* = 3.22–3.66; *p* < 0.001) and LN (*d* = 2.18–2.78; *p* < 0.001). However, there were no differences in absolute fat oxidation between LN and U (*p* = 0.79). Blood lactate was elevated across conditions. Specifically, there were medium (*d* = 0.73) increases in blood lactate with LN vs. U (*p* = 0.026), and large increases with LH vs. U (*d* = 2.88; *p* < 0.001) and LN (*d* = 2.15; *p* < 0.001).

## Discussion

This study investigated the effects of load carriage in normoxia and normobaric hypoxia on ventilatory responses, hemodynamics, tissue oxygenation, and metabolism. Primary findings of this study included the following: (1) load carriage depresses the V_T_ and SV response to exercise matched for absolute VO_2_ in normoxia; (2) increases in Q and V_E_ during normoxic load carriage matched for walking speed and hypoxic load carriage matched for absolute VO_2_ or speed were primarily accomplished via increases in HR and f_B_, respectively; (3) normoxic load carriage increased cerebral O_2_HHb without influencing muscle oxygenation; (4) muscle HHb was increased with hypoxic load carriage matched for absolute VO_2_ and walking speed, but muscle O_2_HHb was only affected when matched for speed; (5) cerebral O_2_HHb and HHb were reduced and increased, respectively, with load carriage in hypoxia; and (6) load carriage did not independently influence substrate utilization when matched for relative and absolute intensities; however, hypoxia increased CHO dependence. The following discussion will focus on matched absolute VO_2_ and walking speed stages for cardiorespiratory responses owing to the predictable nature of findings for exercise of a lower oxygen demand (i.e., matched relative intensity). Discussion of oxygen kinetics and metabolic data will include all intensities.

### Ventilatory responses

Load carriage in normoxia induced a shallower breathing pattern when matched for absolute VO_2_. Specifically, V_T_ and f_B_ were reduced and increased, respectively, relative to U while V_E_ was consistent between normoxic conditions. Comparable alterations in V_T_ and f_B_ have been reported by others investigating the effects of thoracic load carriage (Phillips et al. 2016b; Armstrong et al. 2019), chest wall restriction with inelastic straps (Miller et al. 2002; Tomczak et al. 2011), or fiberglass chest casting (Coast and Cline 2004) and have been attributed to reductions in operating lung volumes (Shei et al. 2017). Few studies have assessed ventilatory mechanics with unloaded exercise compared to load carriage matched for oxygen demand (Phillips et al. 2016b, c; Shei et al. 2018). A study by Phillips et al. (2016b) similarly reported decreases and increases in f_B_ and V_T_, respectively, with prolonged (45 min; ~ 3.0 L min^−1^) load carriage (25 kg). However, differences in V_T_ were not observed until late in exercise (i.e., 35 min), which contrasts with the present study (i.e., 10 min stages; 20 total min of exercise at stage 2 end). Additional distinctions occurring late in exercise were increases in V_E_ and VO_2_ with load carriage, which the authors suggested were likely the result of ventilatory compensation for increases in deadspace ventilation.

An explanation for these discrepancies with Phillips et al. (2016b) is not immediately clear but may be related to methodological differences relating to exercise intensity and ventilatory demand. In a study by Dominelli et al. (2012), the effects of carrying various loads at two fixed workloads on operating lung volumes and the power (i.e., energy requirement) of breathing were investigated. With increasing loads up to 35 kg, end expiratory lung volumes were reduced. Interestingly, the power of breathing was maintained with the heaviest load relative to no load when ventilation was matched at ~ 45  L min^−1^ but increased substantially at higher levels of V_E_ (~ 70 L min^−1^). This finding suggests that the alterations in operating lung volumes or breathing mechanics induced by the heavy load may have been compensatory adaptations permitting the maintenance of the power of breathing and V_E_ at the lower ventilatory demand. However, this altered breathing pattern may have been insufficient to meet ventilatory needs at higher workloads, thus requiring adoption of breathing patterns more consistent with unloaded exercise (e.g., similar or higher V_T_) despite the apparently necessary increase in the power of breathing to achieve it. This theory is supported by ventilatory data from the present study in which a shallower breathing pattern was adopted at approximately the same “low” V_E_ (~ 45 L min^−1^; matched absolute VO_2_ stage) while a similar V_T_ was observed between U and LN when matched for walking speed, an intensity necessitating a higher VO_2_ and V_E_ (~ 70 L min^−1^) in the loaded condition. Importantly, potential increases in the power of breathing at high V_E_ rates may contribute to fatigue of the respiratory muscles during prolonged exercise and deterioration of breathing mechanics over time (e.g., increases and decreases in f_B_ and V_T_, respectively), thereby requiring increases in V_E_ to offset reductions in alveolar ventilation. This hypothesis is supported by the data from Phillips et al. (2016b), which employed exercise resulting in a V_E_ of ~ 75 L min^−1^, which caused respiratory muscle fatigue in the loaded condition and increases in V_E_ over time likely to compensate for reductions in V_T_ late in exercise. More research is clearly warranted to investigate these questions and determine whether a potential threshold exists for V_E_-dependent alterations in breathing mechanics that influence the power of breathing.

The predictable increases in V_E_ with hypoxic load carriage relative to unloaded normoxic exercise were primarily achieved via increases in f_B_. Specifically, when matched for absolute VO_2_, V_T_ and f_B_ were maintained and increased, respectively, relative to U. At the same walking speed, f_B_ was further increased relative to U to meet the increased ventilatory demands. To our knowledge, only one study has investigated the ventilatory responses to load carriage in acute hypoxia. In this study, Hinde et al. (2018) assessed breathing mechanics and respiratory muscle fatigue with load carriage (5.5 km; 18.2 kg) in hypoxia (~ 11.8% F_I_O_2_; ~ 4300 m) or cold (− 10 °C). Similar to the present results for normoxic load carriage, reductions and increases in V_T_ and f_B_, respectively, were observed with load carriage relative to unloaded walking. Interestingly, this effect spanned environmental conditions. However, this study did not control for intensity, allowing subjects to self-select walking speeds, which, while beneficial for ecological validity, prevents easy interpretation of ventilatory responses owing to differences in VO_2_ across conditions. Nevertheless, careful examination of available data from this study in combination with results from the matched oxygen demand and walking speed stages of the present study seem to lend further support for the notion that ventilatory demands may mediate breathing mechanics during load carriage and that this theory also applies to hypoxic conditions. Specifically, Hinde et al. (2018) reported relatively low V_E_ values (≤ 30 L min^−1^) across all conditions and time points coupled with apparent trends for shallower breathing patterns (which may have achieved statistical significance given greater statistical power), in the hypoxic conditions with load vs. unloaded walking at sea level (e.g., 0.82–0.83 vs. 0.87–0.98 and 35–41 vs. 28–34 for V_T_ and f_B_, respectively). In the present study in which V_E_ at matched oxygen demand and walking speed was ~ 55–95 L min^−1^ in the hypoxic condition, V_T_ was either similar or increased relative to U and LN, which again suggests that an increased power of breathing may have been required to achieve this higher V_E_. Importantly, this potential elevation in ventilatory work would likely hasten the development of respiratory muscle fatigue and the attendant metaboreflex (St Croix et al. 2000; Derchak et al. 2002), particularly in hypoxia where diaphragm fatiguability is increased (Babcock et al. 1995; Reinhard et al. 2023). Given this, more research is needed to assess how hypoxia, ventilatory demand, and breathing mechanics may interact to influence respiratory fatigue and blood flow responses.

### Hemodynamic responses

Thoracic load carriage in normoxia altered hemodynamic variables in ways that mirrored ventilatory responses. Specifically, SV and HR were reduced and increased, respectively, while Q was maintained with LN compared to U at the same absolute VO_2_. Miller et al. (2002) observed similar responses for SV and HR when chest wall restriction was induced via inelastic strapping. However, this study also reported a 12% reduction in Q, which was not observed in the present study. It is likely that differences in the degree of chest wall restriction contributed to these conflicting findings for Q. Indeed, inelastic strapping has been reported to reduce total lung capacity and forced vital capacity by 33% and 40%, respectively (Miller et al. 2002; Tomczak et al. 2011), whereas load carriage of comparable mass to the present study reduced forced vital capacity by only 4–12% (Dominelli et al. 2012; Phillips et al. 2016b; Armstrong et al. 2019) and did not affect total lung capacity (Phillips et al. 2016b). This suggests attenuated chest wall restriction with thoracic load carriage, which may permit maintenance of Q. Studies investigating hemodynamic responses to thoracic load carriage report equivocal findings, which are likely due to methodological differences (i.e., type and mass of load, subject characteristics) or confounding variables (i.e., environmental conditions) (Sagiv et al. 2006; Nelson et al. 2009).

Our finding of reduced EDV with LN vs. U at matched oxygen demand suggests attenuated venous return as a likely mechanism for the SV reduction with load. Venous return is influenced by the respiratory muscle pump, which creates negative pressure swings via alternating decreases (i.e., inspiration) and increases (i.e., expiration) in intrathoracic pressures (Miller et al. 2005). Attenuated negative pressure swings have been reported with chest wall restriction via inelastic straps (Miller et al. 2002) and could conceivably impact venous return with thoracic load carriage. Alternatively, reductions in SV may have resulted from increases in sympathetic activation and consequent increases in SVR stemming from a potential respiratory muscle metaboreflex. Indeed, others have reported a metaboreflex-induced increase in SVR with increased respiratory muscle work (Sheel et al. 2001, 2002). Moreover, a number of load carriage studies have observed elevations in arterial blood pressure suggesting elevated sympathetic outflow (Hong et al. 2000; Sagiv et al. 2006; Ribeiro et al. 2014). While blood pressure was not measured in the present study, our finding of similar SVR and muscle oxygenation (discussed below) between U and LN at matched absolute VO_2_ suggests that a respiratory muscle metaboreflex was less likely to be a contributing mechanism. Indeed, Sheel et al. (2002) observed that, with graded increases in inspiratory muscle work, elevations in limb vascular resistance only occurred with contractions sufficient to elicit diaphragm fatigue, which may not have occurred in the present study given the nature/degree of respiratory muscle work (i.e., resulting from thoracic load carriage) and relatively short windows of exercise surrounded by periods of rest.

Interestingly, SV and EDV were maintained and SVR reduced with LH compared to U at matched VO_2_ and walking speeds. An increase in SV with exercise in hypoxia relative to normoxia at these intensities would generally be expected (e.g., with unloaded exercise) as part of the normal cardiovascular compensation for reduced oxygen availability (Naeije 2010). Moreover, reductions in SVR are logical given the vasodilatory response in active muscle to the increased metabolite production (i.e., from higher relative intensities in hypoxia) and oxygen demand (i.e., at matched speed) at these intensities (VanTeeffelen and Segal 2003; Segal and Bearden 2012). Nevertheless, the matching of SV between U and LH is somewhat unexpected given the previously described reduction in SV with load carriage in normoxia. Moreover, prior studies have observed increases and decreases in sympathetic outflow and leg blood flow, respectively, during hypoxic exercise with inspiratory resistance (Katayama et al. 2013) or hypoxemic exercise with resistive breathing (i.e., among COPD patients) (Simon et al. 2001), which would likely increase afterload. While purely speculative, it is possible that any potential respiratory muscle pump-mediated mechanism influencing venous return was abolished by the maintenance of a similar breathing pattern (i.e., matched V_T_) and thus respiratory pump effectiveness with LH compared to U. Alternatively, the relatively short exercise duration may have prevented development of respiratory muscle fatigue sufficient to induce a metaboreflex that could affect SVR. Regardless of the precise mechanism, our findings suggest that load carriage does not compromise Q in either environmental condition when matched for oxygen demand, but it is yet to be determined how hemodynamic responses may be altered by more prolonged load carriage in hypoxia where respiratory muscle fatigue is more likely to occur.

### Oxygen kinetics

To our knowledge, this is the first study to investigate the effects of thoracic load carriage in different environmental conditions on oxygen kinetics. In muscle during normoxic exercise, oxygenation and regional blood flow was similar between unloaded and loaded exercise. Additionally, HHb was reduced with LN compared to the other conditions at matched relative intensity likely owing to less oxygen extraction given the lower oxygen demand. Based on the aforementioned potential for blood flow redistribution via a respiratory muscle metaboreflex (St Croix et al. 2000; Derchak et al. 2002) or the increased use of accessory muscles attendant to load carriage (Holewijn 1990; Devroey et al. 2007), it was predicted that locomotor muscle oxygenation would be reduced by load carriage. These results indicate that load carriage does not impair oxygen delivery to locomotor muscle during exercise. However, more research is needed that assesses blood flow and oxygen kinetics with unloaded and loaded exercise matched for higher intensities in which central factors may be more limiting to oxygenation and exercise performance.

Muscle oxygenation was also mostly maintained with load carriage in hypoxia except at the highest exercise intensity (i.e., matched speed). Additionally, oxygen extraction appeared to be elevated at the higher intensities as evidenced by increases in HHb. Similar levels of muscle oxygenation combined with increased oxygen extraction at matched workloads have been reported by some (Subudhi et al. 2008; Rosales et al. 2022), but not others (Subudhi et al. 2007; Angeli et al. 2019), with hypoxic vs. normoxic exercise. An explanation for this equivocal data may be found in the methodological differences between studies, which include exercise modality, duration, hypoxia type (i.e., normobaric or hypobaric), and muscles assessed. Another interpretation of the present study’s findings, when accounting for the within-condition reduction in oxygenation with LH between matched relative and absolute intensities, and the between-condition reduction with LH compared to the other conditions at matched speed, is that muscle oxygenation seemed to progressively decline with intensity. Such an intensity-dependent reduction in muscle oxygenation has been reported previously (Subudhi et al. 2007, 2008), and may be mediated by regional blood flow. Indeed, tHHb concentrations were increased during LH at matched relative intensity compared to the other conditions, which likely contributed to the aforementioned maintenance of tissue oxygenation. Thereafter, tHHb was similar between conditions, which may have resulted in insufficient blood flow to sustain oxygenation levels (i.e., given likely lower oxygen diffusion and arterial oxygen content) requiring increased oxygen extraction to compensate. Thus, our data indicates that regional blood flow may be limiting for muscle oxygenation at moderate to high intensities in hypoxia, but not normoxia. Of interest, prior studies have reported progressive increases in muscle tHHb with exercise intensity during cycling in either normoxia or hypoxia (Subudhi et al. 2007). In the present study, tHHb did not change during exercise in the LH condition despite progressive increases in intensity. As such, it is tempting to speculate that this may be a load carriage-specific response. However, further study comparing unloaded vs. loaded exercise in hypoxia is needed to confirm this.

Cerebral oxygenation responses indicate a load carriage-specific effect in normoxia. Specifically, cerebral oxygenation was increased at every exercise intensity with load carriage while HHb was similar or lower compared to U. An explanation for this interesting finding is not immediately clear. It seems reasonable to assume that increased oxygenation is the result of increases in blood flow, which might be expected with LN vs. U given: (1) the higher relative exercise intensities and consequent increased sympathetic activation/blood pressure (i.e., at matched oxygen demand) (Hong et al. 2000; Sagiv et al. 2006; Ribeiro et al. 2014) and (2) the increased Q (i.e., at matched speed). Our data mostly supports this notion, as we noted a strong trend (*p* = 0.051) for an increase in tHHb at matched oxygen demand and a statistical increase in tHHb at matched speed. As previously discussed, it is possible that load carriage results in blood flow redistribution stemming from chest wall restriction or increased respiratory muscle work. It is conceivable that these effects may augment cerebral blood flow in some way. However, more research is clearly needed to elucidate the mechanism for this seeming over-compensation in cerebral oxygenation.

Responses to load carriage in hypoxia for cerebral oxygenation were, in some ways, the inverse of those observed during LN. Specifically, O_2_HHb was decreased relative to U or LN at rest and every exercise intensity, and HHb was correspondingly increased. In the case of LH, this seemed to be more clearly mediated by increases in cerebral blood flow, as tHHb was increased vs. U or LN at every exercise intensity. This exaggerated tHHb response with LH may be related to impaired cerebral autoregulation in hypoxia combined with the aforementioned load carriage factors affecting blood flow distribution (Derchak et al. 2002; Miller et al. 2005; Ainslie et al. 2007). Regardless of the mechanism, it appears that load carriage increases cerebral blood flow and that changes in hemoglobin status seem to be mediated by environmental condition. Based on prior studies, this is a novel finding. Others have similarly reported reductions and increases in cerebral O_2_HHb and HHb, respectively, with exercise in hypoxia (Ainslie et al. 2007; Bourdillon et al. 2014; Rosales et al. 2022). However, none of these studies have observed increased tHHb in hypoxia relative to normoxia. It is possible that the observed increase in tHHb was region-specific and not an indication of increases in overall cerebral blood flow. Indeed, others have reported discrepancies between middle cerebral artery blood flow velocity and tHHb in the frontal cortex region (Ainslie et al. 2007; Bourdillon et al. 2014). An additional possibility is that, given the increased biomechanical difficulty of bearing a heavy load (Attwells et al. 2006), frontal cortex motor activity and metabolic demand were increased relative to unloaded exercise thereby enhancing the distribution of cerebral blood flow to that region (Delp et al. 2001), without substantially altering overall cerebral blood flow. Whatever the case may be, our finding has potentially important health implications as cerebral pressure/perfusion has been implicated in the pathophysiology of acute mountain sickness and high-altitude cerebral edema (Hackett 1999; Taylor 2011). Thus, the present study indicates that load carriage exercise may exacerbate the risks for these conditions; although, more study is needed to confirm this.

### Metabolism and perceptual responses

This is the first study to evaluate metabolic responses to load carriage in different environmental conditions and account for the effects of load on relative and absolute exercise intensities. With LN, relative and absolute CHO oxidation were similar to U at the same relative intensity and absolute VO_2_. This aligns with prior studies (Phillips et al. 2016b, c), which reported similar RER values between loaded and unloaded conditions matched for oxygen demand in males and females. When matched for walking speed, LN increased relative/absolute CHO oxidation and lactate vs. U, which also aligns with prior studies (Blacker et al. 2009; Arcidiacono et al. 2023) and is predictable given the higher relative and absolute exercise intensity in the loaded condition (Romijn et al. 1993). Collectively, these data suggest that load carriage does not independently mediate substrate utilization when matched for relative intensity and oxygen demand with unloaded exercise (i.e., at least given the relatively low matched intensities [40–45%VO_2max_] employed in this study) but increases CHO dependence when walking at the same speed.

In hypoxia, absolute and relative CHO oxidation were reduced and maintained, respectively, with LH compared to the normoxic conditions at matched relative intensity. This finding aligns with a recent meta-analysis that evaluated the effects of hypoxia on substrate utilization (Griffiths et al. 2019b). Moreover, it was predictable given the lower oxygen demand and overall substrate oxidation with LH at this intensity. When matched for oxygen demand or walking speed, relative/absolute CHO oxidation and lactate were increased compared to the normoxic conditions. This was also predictable given the higher relative intensities imposed by hypoxia during these stages (~ 60–80 vs. 40–45%VO_2max_) and aligns with prior studies evaluating unloaded exercise (Lundby and Van Hall 2002; Young et al. 2018). Additionally, blood glucose was elevated during LH at all intensities. Prior studies have observed increased rates of glucose appearance and utilization by exercising skeletal muscle in hypoxia (Brooks et al. 1991; Roberts et al. 1996). This glucose dependence in hypoxia has been attributed to the fact that CHO is a more energy efficient fuel than lipids (i.e., yields more energy per liter of oxygen; Mazzeo 2008) and that epinephrine concentrations are increased with hypoxic exposure likely elevating liver glycogenolysis (Mazzeo et al. 1991).

Interestingly, the bioenergetics of walking did not appear to be affected by load carriage despite the above-described cardiorespiratory responses. Specifically, the energy cost of walking (i.e., net above resting) at the same absolute VO_2_ (~ 1.7 L min^−1^) equated to ~ 4.0 J kg^−1^ m^−1^ across conditions (i.e., when accounting for differences in mass [U = 81.7 kg; LN/LH = 111.7 kg], and speed [U = 1.2 m s^−1^; LN/LH = 1.0 m s^−1^ assuming an energy equivalent of 20.9 kJ L^−1^ O_2_^−1^). This value is essentially equal to that reported previously with unloaded walking at a similar speed (~ 1.2 m s^−1^) and gradient (10%) (Minetti et al. 2002). Collectively, this suggests that walking biomechanics and efficiency are maintained with load carriage in normoxia and hypoxia. However, it is unknown whether this still applies at different speeds, grades, and degrees of hypoxia. A study by Phillips et al. (2016a) observed that VO_2_ relative to total mass was similar between unloaded and loaded (25 kg) conditions during constant speed (1.5 m s^−1^) graded exercise testing up to a gradient of 4%, but small increases in oxygen cost occurred in the loaded condition at higher gradients (6–8%). Given this, it is possible that load carriage imposes a narrower range of speeds or relative intensities (Boffey et al. 2019) for optimal biomechanical or metabolic efficiency given different terrain and conditions. Further study is clearly warranted to define these parameters to accurately prescribe paces for populations with occupational load carriage requirements in various environments.

Finally, VO_2max_ and RPE were decreased and increased, respectively, in both loaded conditions relative to U. Smaller magnitude reductions in VO_2peak_ (2.5–3.5%) vs. the present study (7.3%) have been reported previously during graded exercise tests in normoxia with load carriage of similar weight (25 kg). While the reduction in aerobic capacity may be related to any of a number of mechanisms influenced by load carriage/chest wall restriction, the reason for the discrepancy in magnitude between studies is unknown. It is possible that the graded exercise test protocol employed by these prior studies resulted in greater local muscle fatigue (i.e., which we observed during pilot testing of a similar protocol), which prevented achievement of a true max in the loaded condition; but this is purely speculation (Phillips et al. 2016b, c). Reductions in VO_2max_ in hypoxia were expected. However, as in normoxia, the magnitude reduction was slightly larger (~ 33%) than has been reported at similar altitudes/levels of hypoxia previously reported with unloaded exercise (26–27%; Buskirk et al. 1967; Cymerman et al. 1989). While it is possible that this is due to load carriage-specific effects as were observed in normoxia, more research is needed to confirm this. Increases in RPE with load carriage have been reported elsewhere with exercise matched for absolute VO_2_ (Phillips et al. 2016b, c) and walking speed (Blacker et al. 2009; Armstrong et al. 2019), which are logical given the resultant higher relative and absolute intensities, respectively. Yet, a novel finding from the present study is an increase in exertion with load carriage in normoxia or hypoxia when matched for relative intensity. This interesting finding suggests that load carriage increases exertion at all intensities even when accounting for relative fitness, which when taken together with the effects on aerobic capacity and overall metabolism strongly supports prior work indicating that load carriage substantially impairs endurance capacity and performance (Phillips et al. 2016a; Arcidiacono et al. 2023).

### Limitations and conclusions

While this study presents a number of novel findings, it is not without limitations. Specifically, there is controversy regarding the equivalency of normobaric and hypobaric hypoxia on physiological responses (Richalet 2020), and some evidence suggests cardiorespiratory or tissue oxygenation responses may differ based on the type of hypoxia (Loeppky et al. 1997; Angeli et al. 2019; Rosales et al. 2022). However, several confounding factors (e.g., time exposed to altitude vs. hypoxia, placebo effects, etc.) complicate interpretation of this data. While more research is needed to clarify potential differences in the physiological response to each condition, available evidence suggests that normobaric hypoxia provides adequate equivalency to simulate actual altitude, particularly given the generalized scope of the present study. Nevertheless, caution should be exercised when extrapolating data from the present study to actual altitude responses.

In conclusion, load carriage seems to impair cardiorespiratory efficiency when matched for oxygen demand with unloaded exercise. However, these effects are nullified during exercise at higher energy or ventilatory demands such as load carriage matched for walking speed with unloaded exercise or in conditions of hypoxia. These responses indicate a potential threshold for adapted breathing mechanics and hemodynamics that may serve (or be a consequence of) physiological adjustments that maintain the power of breathing at lower exercise intensities despite the chest wall restriction imposed by the load. Interestingly, load carriage does not generally appear to impair muscle oxygenation but may increase regional blood flow to the frontal cortex by an unknown mechanism. This increase in blood flow results in opposing effects on hemoglobin status depending on ambient oxygen levels with increases in O_2_HHb in normoxia and HHb in hypoxia. This clearly requires future investigation given the fact that increased cerebral pressure/perfusion may potentiate altitude illness. Finally, load carriage does not seem to independently influence substrate utilization in normoxia or hypoxia when matched for relative intensity but increases in CHO dependence occur when attempting to match walking speeds with unloaded exercise. Taken together, load carriage provides a substantial physiological challenge that is exacerbated in hypoxia, and compensatory responses appear to be only partially effective at maintaining normal function. As such, occupational health and performance may be compromised with load carriage in hypoxic environments, particularly for long-duration or high-intensity work.

## Data Availability

The datasets generated during and/or analysed during the current study are available from the corresponding author on reasonable request.

## Abbreviations

${\eta }_{p}^{2}$: Partial eta squared
CaO_2_: Arterial oxygen content
CHO: Carbohydrate
EDV: End diastolic volume
EF: Ejection fraction
ESV: End systolic volume
f_B_: Breathing frequency
F_I_O_2_: Fraction of inspired oxygen
HHb: Deoxygenated hemoglobin
HR: Heart rate
HR_max_: Maximal heart rate
MOLLE: Modular lightweight load-carrying equipment
NIRS: Near-infrared spectroscopy
Q: Cardiac output
O_2_HHb: Oxygenated hemoglobin
RER: Respiratory exchange ratio
RPE: Rating of perceived exertion
SD: Standard deviation
SpO_2_: Hemoglobin saturation
SV: Stroke volume
SVR: Systemic vascular resistance
V_E_: Minute ventilation
VO_2_: Oxygen consumption
VO_2max_: Maximal oxygen consumption
V_T_: Tidal volume

## References

[CR1] Ainslie PN, Barach A, Murrell C (2007). Alterations in cerebral autoregulation and cerebral blood flow velocity during acute hypoxia: rest and exercise. Am J Physiol Hear Circ Physiol.

[CR2] Angeli CN, Shute RJ, Slivka DR (2019). Higher muscle tissue oxygenation when exposed to hypobaric hypoxia than normobaric hypoxia. J Hum Muscle Perf Ext Env.

[CR3] Arcidiacono DM, Lavoie EM, Potter AW (2023). Peak performance and cardiometabolic responses of modern US army soldiers during heavy, fatiguing vest-borne load carriage. Appl Erg.

[CR4] Armstrong NCD, Ward A, Lomax M (2019). Wearing body armour and backpack loads increase the likelihood of expiratory flow limitation and respiratory muscle fatigue during marching. Ergonomics.

[CR5] Attwells R, Birell S, Hooper R, Mansfield N (2006). Influence of carrying heavy loads on soldiers’ posture, movements and gait. Ergonomics.

[CR6] Babcock MA, Johnson BD, Pegelow DF (1995). Hypoxic effects on exercise-induced diaphragmatic fatigue in normal healthy humans. J Appl Physiol.

[CR7] Blacker SD, Fallowfield JL, Bilzon JLJ, Willems MET (2009). Physiological responses to load carriage during level and downhill treadmill walking. Med Sport.

[CR8] Boffey D, Harat I, Gepner Y (2019). The physiology and biomechanics of load carriage performance. Mil Med.

[CR9] Borg GA (1982). Psychophysical bases of perceived exertion. Med Sci Sport Exerc.

[CR10] Bourdillon N, Fan JL, Kayser B (2014). Cerebral oxygenation during the Richalet hypoxia sensitivity test and cycling time-trial performance in severe hypoxia. Eur J Appl Physiol.

[CR11] Boushel R, Piantadosi CA (2000). Near-infrared spectroscopy for monitoring muscle oxygenation. Acta Physiol Scand.

[CR12] Brooks GA, Butterfield GE, Wolfe RR (1991). Increased dependence on blood glucose after acclimatization to 4,300 m. J Appl Physiol.

[CR13] Buskirk E, Kollias J, Akers R, Prokop E (1967). Maximal performance at altitude and on return from altitude in conditioned runners. J Appl Physiol.

[CR14] Coast JR, Cline CC (2004). The effect of chest wall restriction on exercise capacity. Respirology.

[CR15] Cohen J (1973). Eta-squared and partial eta-squared in fixed factor anova designs. Educ Psychol Meas.

[CR16] Cohen J (2013). Statistical power analysis for the behavioral sciences.

[CR17] Conkin J (2011). PH_2_O and simulated hypobaric hypoxia. Aviat Sp Environ Med.

[CR18] Cymerman A, Reeves JT, Sutton JR (1989). Operation Everest II: maximal oxygen uptake at extreme altitude. J Appl Physiol.

[CR19] Dean C (2004) The modern warrior’s combat load. Dismounted operations in Afghanistan April–May 2003. Fort Leavenworth

[CR20] Delp M, Armstrong R, Godfrey D (2001). Exercise increases blood flow to locomotor, vestibular, cardiorespiratory and visual regions of the brain in miniature swine. J Physiol.

[CR21] Dempsey JA, Romer L, Rodman J (2006). Consequences of exercise-induced respiratory muscle work. Respir Physiol Neurobiol.

[CR22] Derchak AP, Sheel WA, Morgan BJ, Dempsey JA (2002). Effects of expiratory muscle work on muscle sympathetic nerve activity. J Appl Physiol.

[CR23] Devroey C, Jonkers I, de Becker A (2007). Evaluation of the effect of backpack load and position during standing and walking using biomechanical, physiological and subjective measures. Ergonomics.

[CR24] Dominelli PB, Sheel AW, Foster GE (2012). Effect of carrying a weighted backpack on lung mechanics during treadmill walking in healthy men. Eur J Appl Physiol.

[CR25] Drain J, Billing D, Neesham-Smith D, Aisbett B (2016). Predicting physiological capacity of human load carriage—a review. Appl Erg.

[CR26] Duncan A, Meek JH, Clemence M (1995). Optical pathlength measurements on adult head, calf and forearm and the head of the newborn infant using phase resolved optical spectroscopy. Phys Med Biol.

[CR27] Faghy MA, Brown PI (2014). Thoracic load carriage-induced respiratory muscle fatigue. Eur J Appl Physiol.

[CR28] Faghy M, Blacker S, Brown PI (2016). Effects of load mass carried in a backpack upon respiratory muscle fatigue. Eur J Sport Sci.

[CR29] Faghy MA, Shei RJ, Armstrong NCD (2022). Physiological impact of load carriage exercise: current understanding and future research directions. Physiol Rep.

[CR30] Ferretti G, Moia C, Thomet JM, Kayser B (1997). The decrease of maximal oxygen consumption during hypoxia in man: a mirror image of the oxygen equilibrium curve. J Physiol.

[CR31] Goodman J, Hassell K, Irwin D (2014). The splenic syndrome in individuals with sickle cell trait. High Alt Med Biol.

[CR32] Griffiths A, Deighton K, Shannon OM (2019). Substrate oxidation and the influence of breakfast in normobaric hypoxia and normoxia. Eur J Appl Physiol.

[CR33] Griffiths A, Shannon OM, Matu J (2019). The effects of environmental hypoxia on substrate utilisation during exercise: a meta-analysis. J Int Soc Sport Nutr.

[CR34] Hackett PH (1999). High altitude cerebral edema and acute mountain sickness. Hypoxia.

[CR35] Hinde K, Low C, Lloyd R, Cooke C (2018). Interaction between ambient temperature, hypoxia, and load carriage on respiratory muscle fatigue. Aerosp Med Hum Perform.

[CR36] Hinde KL, Low C, Lloyd R, Cooke CB (2020). Inspiratory muscle training at sea level improves the strength of inspiratory muscles during load carriage in cold-hypoxia. Ergonomics.

[CR37] Holewijn M (1990). Physiological strain due to load carrying. Eur J Appl Physiol.

[CR38] Holewun M, Lotens WA (2007). The influence of backpack design on physical performance. Ergonomics.

[CR39] Hong Y, Li JX, Wong ASK, Robinson PD (2000). Effects of load carriage on heart rate, blood pressure and energy expenditure in children. Ergonomics.

[CR40] Howley ET, Bassett DR, Welch HG (1995). Criteria for maximal oxygen uptake: review and commentary. Med Sci Sport Exerc.

[CR41] Jeukendrup AE, Wallis GA (2005). Measurement of substrate oxidation during exercise by means of gas exchange measurements. Int J Sport Med.

[CR42] Katayama K, Yamashita S, Ishida K (2013). Hypoxic effects on sympathetic vasomotor outflow and blood pressure during exercise with inspiratory resistance. Am J Physiol Reg Int Comp Physiol.

[CR43] Knapik J, Staab J, Bahrke M (1991). Soldier performance and mood states following a strenuous road march. Mil Med.

[CR44] Knapik JJ, Reynolds KL, Harman E (2004). Soldier load carriage: historical, physiological, biomechanical, and medical aspects. Mil Med.

[CR45] Loeppky JA, Icenogle M, Scotto P (1997). Ventilation during simulated altitude, normobaric hypoxia and normoxic hypobaria. Resp Physiol.

[CR46] Looney DP, Santee WR, Karis AJ (2018). Metabolic costs of military load carriage over complex terrain. Mil Med.

[CR47] Lundby C, Van Hall G (2002). Substrate utilization in sea level residents during exercise in acute hypoxia and after 4 weeks of acclimatization to 4100 m. Acta Physiol Scand.

[CR48] Mazzeo RS (2008). Physiological responses to exercise at altitude: an update. Sport Med.

[CR49] Mazzeo RS, Bender PR, Brooks GA (1991). Arterial catecholamine responses during exercise with acute and chronic high-altitude exposure. Am J Physiol.

[CR50] Miller JD, Beck KC, Joyner MJ (2002). Cardiorespiratory effects of inelastic chest wall restriction. J Appl Physiol.

[CR51] Miller JD, Pegelow DF, Jacques AJ, Dempsey JA (2005). Skeletal muscle pump versus respiratory muscle pump: modulation of venous return from the locomotor limb in humans. J Physiol.

[CR52] Minetti AE, Moia C, Roi GS (2002). Energy cost of walking and running at extreme uphill and downhill slopes. J Appl Phys.

[CR53] Naeije R (2010). Physiological adaptation of the cardiovascular system to high altitude. Prog Cardiovasc Dis.

[CR54] Neary JP (2004). Application of near infrared spectroscopy to exercise sports science. Can J Appl Physiol.

[CR55] Nelson MD, Haykowsky MJ, Mayne JR (2009). Effects of self-contained breathing apparatus on ventricular function during strenuous exercise. J Appl Physiol.

[CR56] Pandolf KB, Givoni B, Goldman RF (1977). Predicting energy expenditure with loads while standing or walking very slowly. J Appl Physiol Respir Environ Exerc Physiol.

[CR57] Phillips DB, Stickland MK, Lesser IA, Petersen SR (2016). The effects of heavy load carriage on physiological responses to graded exercise. Eur J Appl Physiol.

[CR58] Phillips DB, Stickland MK, Petersen SR (2016). Ventilatory responses to prolonged exercise with heavy load carriage. Eur J Appl Physiol.

[CR59] Phillips DB, Stickland MK, Petersen SR (2016). Physiological and performance consequences of heavy thoracic load carriage in females. Appl Physiol Nutr Metab.

[CR60] Pihlainen K, Santtila M, Häkkinen K (2014). Cardiorespiratory responses induced by various military field tasks. Mil Med.

[CR61] Reinhard PA, Archiza B, Welch JF (2023). Effects of hypoxia on exercise-induced diaphragm fatigue in healthy males and females. Physiol Rep.

[CR62] Ribeiro F, Oliveira NL, Pires J (2014). Treadmill walking with load carriage increases aortic pressure wave reflection. Rev Port Cardio.

[CR63] Richalet J-P (2020). CrossTalk opposing view: barometric pressure, independent of PO2, is not the forgotten parameter in altitude physiology and mountain medicine. J Physiol.

[CR64] Roberts AC, Reeves JT, Butterfield GE (1996). Altitude and beta-blockade augment glucose utilization during submaximal exercise. J Appl Physiol.

[CR65] Romijn JA, Coyle EF, Sidossis LS (1993). Regulation of endogenous fat and carbohydrate metabolism in relation to exercise intensity and duration. Am J Physiol Endocrinol Metab.

[CR66] Rosales AM, Shute RJ, Hailes WS (2022). Independent effects of acute normobaric hypoxia and hypobaric hypoxia on human physiology. Sci Rep.

[CR67] Sagiv M, Sagiv M, Amir R, Ben-Sira D (2006). Left ventricular systolic function during treadmill walking with load carriage in adolescents. J Sport Sci Med.

[CR68] Santee WR, Allison WF, Blanchard LA, Small MG (2001). A proposed model for load carriage on sloped terrain. Aviat Sp Environ Med.

[CR69] Segal SS, Bearden S, Farrell PA, Joyner MJ, Caiozzo VJ (2012). Organization and control of circulation to skeletal muscle. ACSM’s advanced exercise physiology.

[CR70] Shannon OM, Duckworth L, Barlow MJ (2017). Effects of dietary nitrate supplementation on physiological responses, cognitive function, and exercise performance at moderate and very-high simulated altitude. Front Physiol.

[CR71] Sheel AW, Derchak PA, Morgan BJ (2001). Fatiguing inspiratory muscle work causes reflex reduction in resting leg blood flow in humans. J Physiol.

[CR72] Sheel AW, Derchak PA, Pegelow DF, Dempsey JA (2002). Threshold effects of respiratory muscle work on limb vascular resistance. Am J Physiol Hear Circ Physiol.

[CR73] Shei R-J, Chapman RF, Gruber AH, Mickleborough TD (2017). Respiratory effects of thoracic load carriage exercise and inspiratory muscle training as a strategy to optimize respiratory muscle performance with load carriage. Springer Sci Rev.

[CR74] Shei RJ, Chapman RF, Gruber AH, Mickleborough TD (2018). Inspiratory muscle training improves exercise capacity with thoracic load carriage. Physiol Rep.

[CR75] Simon M, LeBlanc P, Jobin J (2001). Limitation of lower limb VO(2) during cycling exercise in COPD patients. J Appl Physiol.

[CR76] Sol JA, Ruby BC, Gaskill SE (2018). Metabolic demand of hiking in wildland firefighting. Wilderness Environ Med.

[CR77] St Croix CM, Morgan BJ, Wetter TJ, Dempsey JA (2000). Fatiguing inspiratory muscle work causes reflex sympathetic activation in humans. J Physiol.

[CR78] Subudhi AW, Dimmen AC, Roach RC (2007). Effects of acute hypoxia on cerebral and muscle oxygenation during incremental exercise. J Appl Physiol.

[CR79] Subudhi AW, Lorenz MC, Fulco CS, Roach RC (2008). Cerebrovascular responses to incremental exercise during hypobaric hypoxia: effect of oxygenation on maximal performance. Am J Physiol Hear Circ Physiol.

[CR80] Taylor AT (2011). High-altitude illnesses: physiology, risk factors, prevention, and treatment. Rambam Maimonides Med J.

[CR81] Tomczak SE, Guenette JA, Reid WD (2011). Diaphragm fatigue after submaximal exercise with chest wall restriction. Med Sci Sport Exerc.

[CR82] TRADOC (2022) Foot Marches ATP 3-21.18

[CR83] Van Beekvelt MCP, Colier WNJM, Wevers RA, Van Engelen BGM (2001). Performance of near-infrared spectroscopy in measuring local O(2) consumption and blood flow in skeletal muscle. J Appl Physiol.

[CR84] VanTeeffelen JWGE, Segal SS (2003). Interaction between sympathetic nerve activation and muscle fibre contraction in resistance vessels of hamster retractor muscle. J Physiol.

[CR85] Young AJ, Berryman CE, Kenefick RW (2018). Altitude acclimatization alleviates the hypoxia-induced suppression of exogenous glucose oxidation during steady-state aerobic exercise. Front Physiol.

[CR86] Young AJ, Reeves JT (2002) Human adaptation to high terrestrial altitude. In: Medical aspects of harsh environments. pp 647–691

